# Prevalence and determinants of recurrent laryngeal nerve injury after thyroidectomy: a Systematic Review and meta-analysis

**DOI:** 10.3389/fendo.2026.1764332

**Published:** 2026-04-29

**Authors:** Rifat Awawda, Shlomo Merchavy, Uday Abd Elhadi, Alaa Safia

**Affiliations:** 1 Emek Medical Center, Afula, Israel; 2 Department of Otolaryngology, Head & Neck Surgery Unit, Rebecca Ziv Medical Center, Safed, Israel

**Keywords:** intraoperative nerve monitoring, meta-analysis, recurrent laryngeal nerve injury, RLNI, thyroidectomy

## Abstract

**Background:**

Recurrent laryngeal nerve injury (RLNI) is a major complication of thyroid surgery and may lead to voice impairment and reduced quality-of-life.

**Aim:**

This systematic review and meta-analysis sought to determine the frequency of temporary/permanent RLNI after different types of thyroid operations and to identify surgical and patient-related factors that influence risk.

**Methods:**

PubMed, Scopus, Web of Science, Cochrane Library, and Google Scholar were searched in July 17, 2024. Eligible studies reported original data on RLNI after any type or approach of thyroidectomy in ≥20 patients. Risk of bias using RoB-2 for randomized trials and the Newcastle–Ottawa Scale for observational studies was done. Random-effects meta-analyses were performed. Subgroup and meta-regression analyses explored the effects of surgical type, method, approach, intraoperative nerve monitoring (IONM), hemostasis, drain use, and patient age on RLNI risk.

**Results:**

A total of 199 studies (304,352 patients) were analyzed. Transient RLNI was most frequent after completion thyroidectomy (10%, 95%CI, 4–16%) and hemithyroidectomy (8%, 95%CI, 1–15%), and least after near-total thyroidectomy (2%, 95%CI, 1–3%). Transoral and transcervical approaches had the highest transient RLNI rates (5%, 95%CI, 3–6%), while transaxillary had the lowest (1%, 95%CI, 1–2%). Permanent RLNI was highest in secondary thyroidectomy (2%, 95%CI, 1–3%). Meta-regression identified surgical approach, IONM use, hemostasis method, drain use, and patient age as significant determinant.

**Conclusions:**

Completion and revision surgeries, along with certain surgical approaches, increase RLNI risk. Tailoring surgical technique and optimizing intraoperative strategies are essential to minimize nerve injury.

**Systematic review registration:**

https://www.crd.york.ac.uk/prospero/, identifier CRD42024556259.

## Introduction

Recurrent laryngeal nerve injury (RLNI) is one of the most feared complications of thyroid surgery, with potential consequences including vocal cord paralysis, dysphonia, and airway obstruction (1). The incidence of RLNI varies widely in the literature, largely depending on the definition of nerve injury, the type of surgery, and the use of intraoperative nerve monitoring (IONM) (2–4). While transient RLN injuries often resolve spontaneously, permanent injuries can result in long-term morbidity and significantly impair a patient’s quality of life (5). Thus, understanding the factors that contribute to RLNI is critical for improving surgical outcomes and minimizing postoperative complications.

Thyroidectomy, whether performed for benign or malignant thyroid diseases, remains the most common procedure associated with RLNI (6). The risk of RLNI is influenced by several variables, including the extent of surgery (7, 8), surgeon experience (8), and the use of adjunctive technologies such as IONM (9). Studies have also highlighted the impact of surgical approach—such as open, endoscopic, or robotic—and patient-related factors such as age, gender, and the presence of comorbidities (10–17). However, despite advancements in surgical techniques, RLNI remains a persistent concern, particularly in complex cases such as completion or revision thyroidectomy.

The variability in reported RLNI rates across different studies highlights the need for a systematic evaluation of the factors that influence nerve injury risk. Previous systematic reviews have provided valuable insights into the incidence of RLNI (3, 4, 18, 19), but there remains a lack of comprehensive analysis addressing the interplay between various patient- and surgery-related factors. Moreover, the role of newer surgical techniques and technologies in mitigating RLNI risk has not been adequately explored.

This study aims to fill this gap by conducting a systematic review and meta-analysis of transient and permanent RLNI in patients undergoing thyroidectomy. By analyzing a wide range of patient-related and surgical factors, including surgical approach, method of hemostasis, use of drains, and IONM, we seek to provide a nuanced understanding of RLNI risk.

## Methods

### Design and literature search

The study protocol was registered on PROSPERO. This work was done following the PRISMA (20) (Preferred Reporting Items for Systematic Reviews and Meta-Analyses) and AMSTAR (21) (Assessing the methodological quality of systematic reviews) Guidelines. The review protocol was registered on PROSPERO on June 8, 2024 (Registration number: CRD42024556259). We searched PubMed, Scopus, Web of Science, Cochrane Library, and Google Scholar (first 200 records) (22) up to July 17, 2024. The search strategy, outlined in **Supplementary Table 1**, was adjusted per searched databases. Citations were filtered based on their titles and abstracts. No restrictions were applied regarding the original language of publication. Manual searches included reviewing reference lists and related articles on PubMed (23) as well as on Google Software.

This study was a systematic review and meta-analysis of previously published studies and did not involve any new patient recruitment or direct human subject research. Therefore, institutional review board (IRB) approval was not required. According to institutional policy, this project was deemed exempt from ethical review, and informed consent was waived.

### Selection strategy

Studies were selected using the PICOS framework (24).

The inclusion criteria was as follows:

1. Population: patients undergoing thyroidectomy
2. Intervention: thyroidectomy regardless of the extent or approach
3. Comparison: None
4. Outcome: The rate of transient/permanent RLNI
5. Study Design: All original, observational or experimental studies with >20 cases

The exclusion criteria included the following:

1. Non-original research.
2. Abstract-only publications
3. Case reports or series < 20 cases
4. Duplicated records or studies with overlapping datasets (similar samples and baseline characteristics even if author list differs)
5. Studies combining thyroid and parathyroid surgeries without stratifying the data for the thyroidectomy group alone
6. Studies not reporting RLNI as an outcome
7. Studies reporting invasion of the RLN by thyroid cancer at baseline
8. Animal studies
9. Studies focusing on irrelevant outcomes (interventional, EMG, or diagnostic accuracy studies)

### Data collection and outcomes

The senior author designed the data collection sheet using Microsoft Excel. The sheet was modified four times to fit the data reported by included studies. The final sheet comprised of 4 parts. The first part covered study-related data (authors’ names, year of publication/investigation, country of investigation, study design, sample size, and follow-up period). The second part covered patient-related data (including age, gender, surgical indication, final pathology, neuromonitoring through IONM, revision/secondary surgery, the use of surgical drains and the time to remove them, preoperative laryngoscopic examination, lymphadenectomy, central neck dissection (CND), method of stopping bleeding, type/approach of thyroidectomy, and the experience level of performing surgeons. The third part included the outcome data (transient and permanent RLNI). The fourth part included the methodological quality assessment part. Noteworthy, some studies were translated from Croatian (25), German (26), and Iranian (27) to English.

### Risk of bias assessment

The risk of bias (RoB) of included randomized controlled trials (RCTs) was performed using the revised Cochrane’s RoB-2 tool. For observational studies, the Newcastle Ottawa Scale (NOS) was used. An overall rating of poor (<6 points), fair (6–8 points), or good quality (>8 points) was given.

### Statistical analysis

All analyses were performed using STATA (Version 18, StataCorp, USA) following the in-priori analysis plan. Key transformations included converting medians to means using validated formulas (28, 29). Given the highly heterogeneous sample included in this review, a pooled meta-analysis was deemed inappropriate. Therefore, we conducted subgroup meta-analyses to determine the rates of transient/permanent RLNI based on various patient-related and surgery-related covariates, including surgery type, surgical approach, surgical method, surgical drain use, IONM use, surgeon type, final pathology, homeostasis method, gender, preoperative routine laryngoscopy, and CND.

We employed a random-effects model and used the last observation carried forward method to handle data heterogeneity and minimize missing data risks (30). Heterogeneity was quantified using the I^2^ statistic, with significant heterogeneity defined as I^2^>40% (31). Sensitivity analyses tested the robustness of results with Galbraith plots identifying outliers, and publication bias was assessed with funnel plots and asymmetry tests (32). Funnel plot asymmetry was assessed; however, its interpretation is inherently limited in meta-analyses of proportions due to the mathematical relationship between the effect size and its variance, as well as the presence of substantial heterogeneity.

Multivariate, adjusted meta-regression models were designed to assess the impact of study-level covariates, adjusting for multicollinearity, which was evaluated using variance inflation factors (>5 indicates problematic multicollinearity) (33). The reference group in categorical covariates was selected based on commonality (the most frequently reported subgroup). We did not investigate gender as a covariate, instead, we used the male/female ratio given the fact that most studies did not stratify outcome data based on specific genders. Additionally, the indications for thyroid surgery could not be investigated as included studies did not stratify the outcome data in different indication groups (i.e., Graves’ disease, Hashimoto, thyroid cancer, benign/toxic nodular goiter, etc.). Model fit was assessed with the adjusted R-squared (higher values reflect better fit). Ten studies were needed to run subgroup and meta-regression analyses (significant heterogeneity was mandatory) (34).

## Results

**Figure 1:**
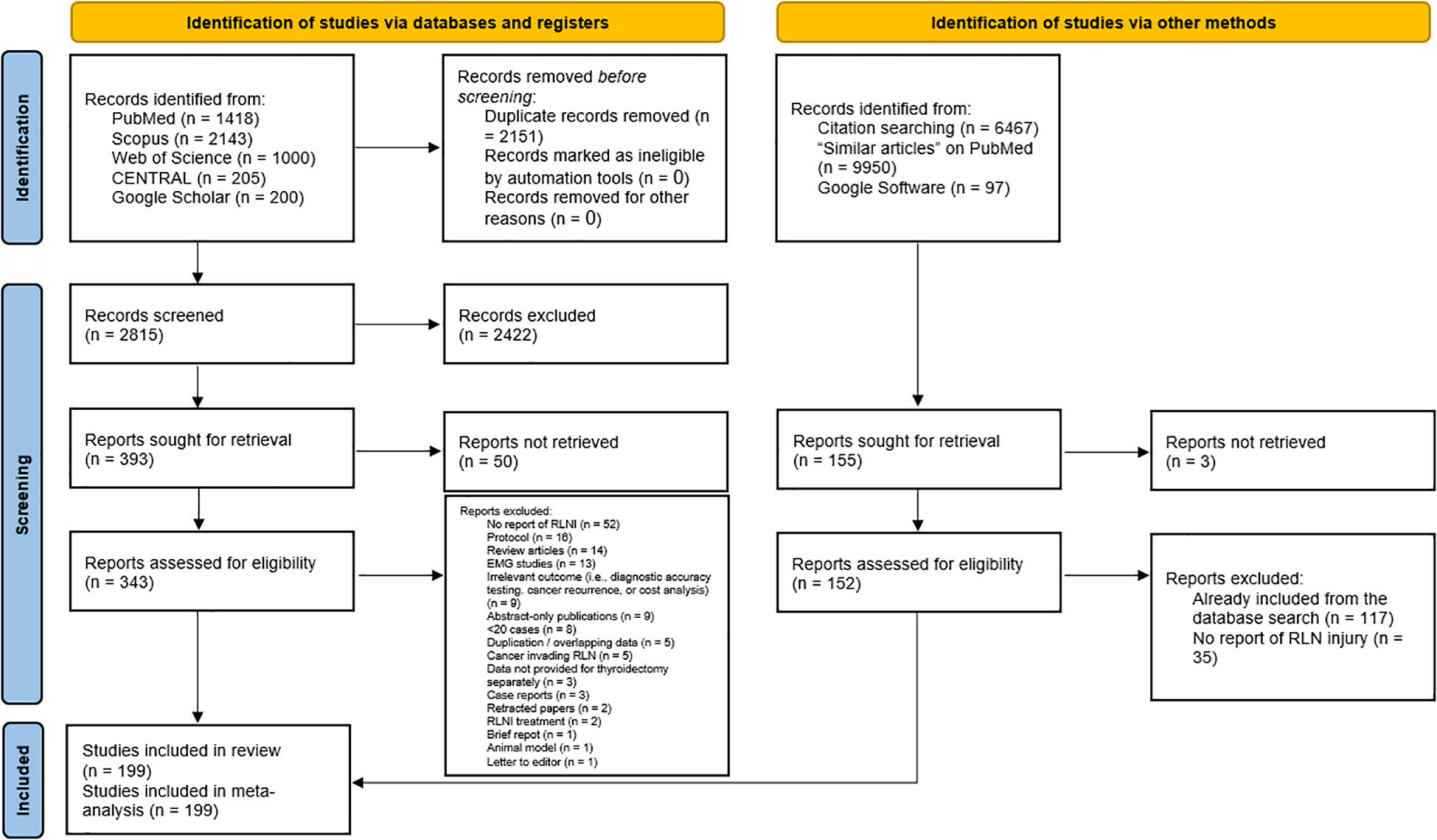
A PRISMA flow diagram showing the results of the literature search and screening process.

Of 4,966 identified records, 2,151 duplicates were removed (**Figure 1**). Title and abstract screening of 2,815 citations excluded 2,422 articles. Full-text review was conducted for 343 articles; 51 were unavailable despite multiple attempts to contact authors. A total of 144 articles were excluded for predefined reasons. Manual screening of 15,514 citations yielded no additional eligible studies. Ultimately, 199 studies were included in the analysis. A full list of included studies can be found in **Supplementary Table 2**.

These studies included 304,352 patients who underwent thyroidectomy. Most studies were retrospective cohorts (71.2%), followed by prospective cohorts (14.6%), randomized trials (12.2%), and other designs. The most common study settings were Italy (9.5%), China (9.0%), Turkey (9.0%), and the United States (8.5%) (**Supplementary Table 3**). Follow-up durations ranged from 1 day to 37 months; 6 months was the most commonly reported time point.

Among 223,610 patients for whom sex was reported, 78.6% were female. Benign disease outcomes were reported in 36 studies (50.7%) and malignant disease in 33 studies (46.5%). Total thyroidectomy was the most commonly investigated procedure (38.5%). IONM use was reported in 40.5% of studies, drains in 10.5%, and routine preoperative laryngoscopy in 25.6%. Central and lateral neck dissections were performed in 14.6% and 5.0% of studies, respectively. Consultant surgeons performed 77.8% of operations when surgeon role was specified.

### Methodological quality

Of 24 randomized trials, 18 were rated low risk of bias and 6 had some concerns, largely due to absence of a published protocol. Among observational studies, 71 were rated high quality, 95 fair, and 9 poor (**Supplementary Table 4****;****Supplementary Figure 1**). The definition criteria used to define both transient and permanent RLNI are illustrated in.

### Transient RLNI

**Figure 2:**
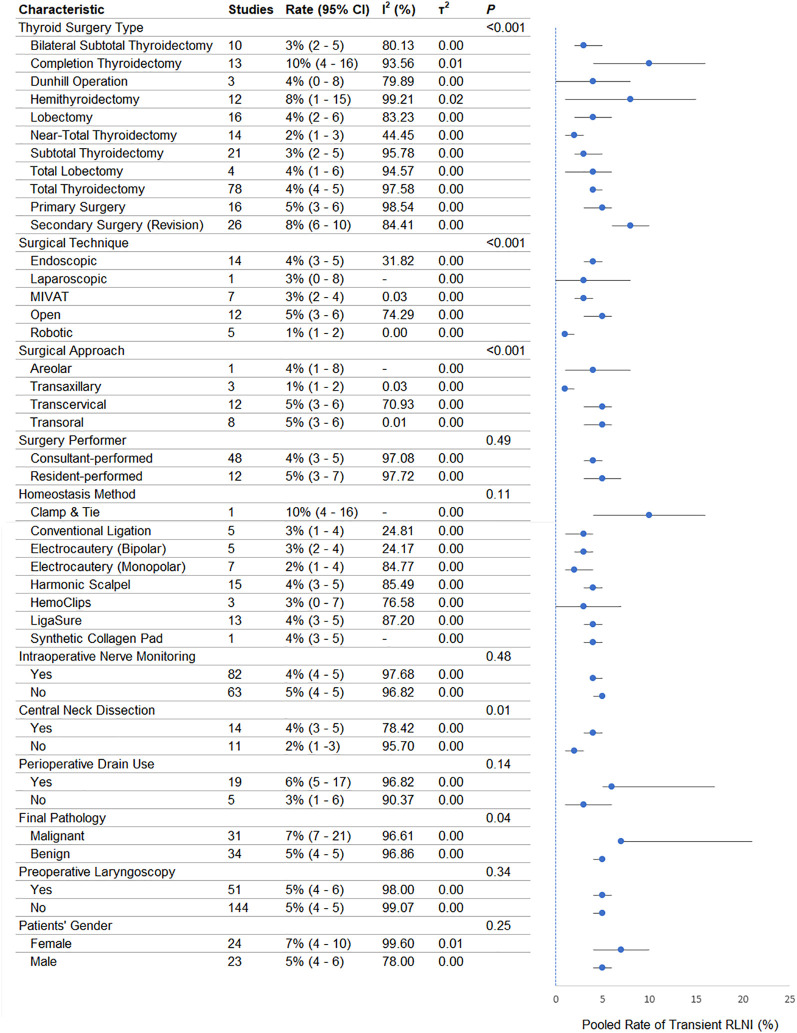
Forest plot showing the pooled rate of transient recurrent laryngeal nerve injury (RLNI) stratified by patient- and surgery-related characteristics. For each subgroup, the pooled rate is presented as a proportion (%) with corresponding 95% confidence intervals (CI), calculated using a random-effects model. The size of each marker reflects the weight of the subgroup, and horizontal lines indicate the 95% CI. The I² statistic represents the degree of heterogeneity within each subgroup, and τ² reflects between-study variance. The p-values reported in the first row of each category correspond to tests for subgroup differences (Q-test), indicating whether there is a statistically significant difference in pooled RLNI rates across subgroups within that category.

A summary of the pooled meta-analytic estimates of transient RLNI based on patients’ and surgical characteristics is provided in **Figure 2**. Surgical type was a significant effect modifier (P = .001). Completion thyroidectomy (10% [95% CI, 4%-16%]) and secondary surgery (8% [6%-10%]) were associated with higher transient RLNI rates than primary thyroidectomy (5% [3%-6%]). Near-total thyroidectomy had the lowest rate (2% [1%-3%]). Surgical method also significantly modified risk (P = .001). Open surgery had the highest RLNI rate (5% [3%-6%]), while robotic surgery had the lowest (1% [1%-2%]). Minimally-invasive video-assisted thyroidectomy (MIVAT) and laparoscopic methods had intermediate rates (3% [2%-4%]). Surgical approach was also significant (P = .001). Transoral and transcervical approaches both had RLNI rates of 5% (95% CI, 3%-6%), while the transaxillary approach had the lowest rate (1% [1%-2%]). Surgeon type did not significantly affect transient RLNI risk (P = .49), with comparable rates between consultants (4%) and residents (5%).

The hemostasis method was not a significant modifier (P = .49). Clamp-and-tie had the highest rate (10% [4%-16%]) but was reported in only one study. Other techniques, including Harmonic scalpel, LigaSure, electrocautery, and collagen pads, had rates between 3% and 4%. IONM use did not significantly affect transient RLNI risk (P = .48). The RLNI rate was 4% with IONM and 5% without. Drain placement was not a significant modifier (P = .14). However, drain use was associated with a higher RLNI rate (6% [5%-17%]) compared with no drains (3% [1%-6%]). Routine preoperative laryngoscopy was also not significant (P = .34), with both groups showing similar RLNI rates (5%). Central neck dissection was associated with increased transient RLNI (P = .01). CND cases had a 4% rate versus 2% without CND.

Final pathology was a significant modifier (P = .04). Malignant pathology was associated with a higher RLNI rate (7% [7%-21%]) than benign (5% [4%-5%]). Sex was not a significant factor (P = .16); RLNI was observed in 7% of females and 5% of males.

In meta-regression (**Table 1**), surgical approach (P < .001), IONM (P = .002), surgical drains (P = .011), and electrocautery use (P = .012) were independently associated with transient RLNI risk. Other variables, including patient age, sex ratio, follow-up duration, CND, surgeon type, and surgery type, were not significant. The model fit was optimal (R² = 100%; I² = 0.01%).

**Table 1:** Adjusted meta-regression analysis of determinants of transient recurrent laryngeal nerve injury during thyroid surgery.

	Coefficient	SE	Z	P-value	5% CI	95% CI
Study design [reference group: RCT] – 192 studies						
Prospective Cohort	0.0029	0.0106	0.2800	0.7800	-0.0178	0.0237
Retrospective Cohort	0.0000	(omitted)	0.0000	0.0000	0.0000	0.0000
Surgical approach [reference group: endoscopic] – 28 studies						
MIVAT	-0.0417	0.0086	-4.8200	0.0000	-0.0586	-0.0247
**Follow-up** (per month) – 96 studies	-0.0014	0.0016	-0.8200	0.4110	-0.0046	0.0019
**Final Pathology** (Malignant vs. Not) – 85 studies	-0.0029	0.0022	-1.2900	0.1960	-0.0072	0.0015
**Final Pathology** (Benign vs. Not) – 85 studies	-0.0034	0.0022	-1.5400	0.1220	-0.0078	0.0009
**IONM** [Reference Group: None] – 193 studies	-0.0310	0.0101	-3.0600	0.0020	-0.0508	-0.0112
**Age** (per year) – 67 studies	0.0014	0.0008	1.8100	0.0710	-0.0001	0.0030
**M/F Ratio – 165 studies**	0.0045	0.0036	1.2400	0.2140	-0.0026	0.0116
**Surgical Drain Use** [Reference Group: None] – 193 studies	0.0350	0.0138	2.5400	0.0110	0.0080	0.0620
**Routine Preoperative Laryngoscopy** [Reference Group: None] – 193 studies	-0.0071	0.0101	-0.7100	0.4810	-0.0269	0.0126
**CND** [Reference Group: None] – 28 studies	0.0002	0.0004	0.4500	0.6520	-0.0007	0.0011
Homeostasis method [reference group: conventional ligation] – 193 studies						
Harmonic Scalpel	-0.0040	0.0059	-0.6700	0.5020	-0.0157	0.0077
LigaSure	-0.0076	0.0074	-1.0200	0.3090	-0.0222	0.0070
Electrocautery	-0.0135	0.0054	-2.5100	0.0120	-0.0241	-0.0030
HemoClips	-0.0325	0.0249	-1.3000	0.1930	-0.0814	0.0164
**Completion Thyroidectomy** [Reference Group: None] – 30 studies	0.0031	0.0059	0.5300	0.5960	-0.0084	0.0147
**Secondary/Revision Thyroidectomy** [Reference Group: Primary Thyroidectomy] – 193 studies	-0.0127	0.0989	-0.1300	0.8970	-0.2066	0.1811
**Resident-Performed Surgery** [Reference Group: Consultant-Performed**] – 18 studies**	-0.0151	0.0321	-0.4700	0.6380	-0.0781	0.0478
**constant**	0.0262	0.0229	1.1400	0.2520	-0.0187	0.0711

Some covariates were excluded from this model either because of insufficient observations (i.e., surgery type) or omitted due to high multicollinearity, defined with variance inflation factor > 10. SE, standard error; CI, confidence interval; CND, central neck dissection; LND, lateral neck dissection; M/F, male/female ratio; IONM, intraoperative nerve monitoring; MIVAT, minimally-invasive video-assisted thyroidectomy; RCT, randomized controlled trial. The bold values indicate the most significant values within each category.

### Permanent RLNI

**Figure 3:**
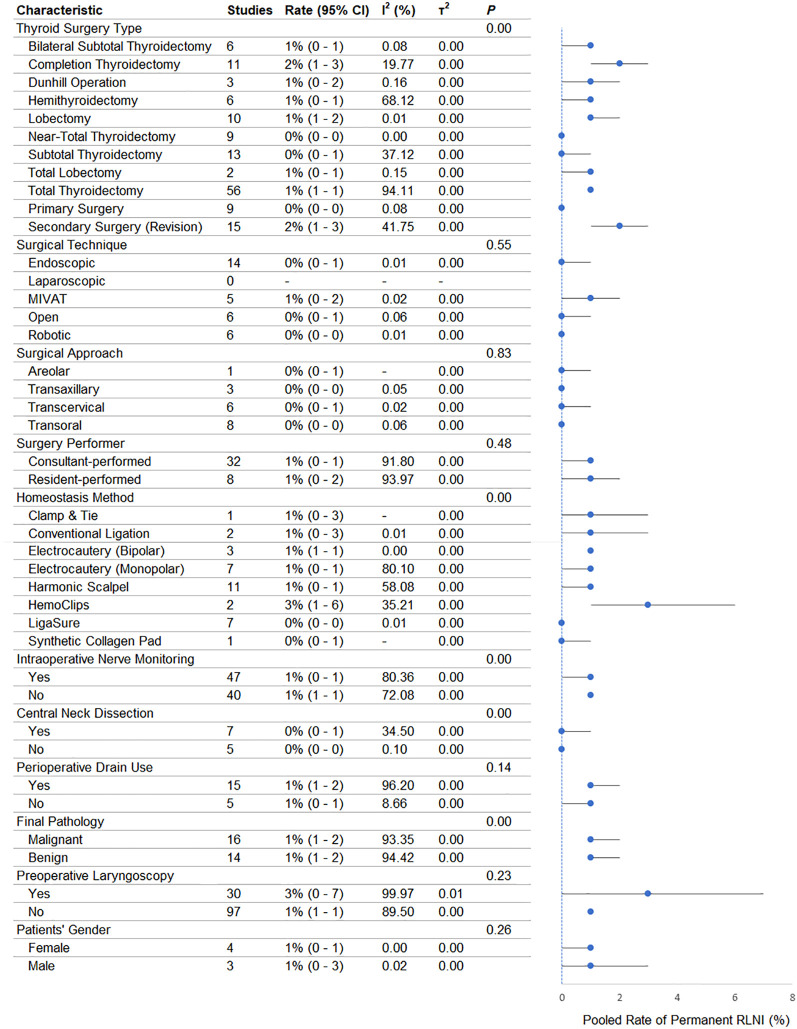
Forest plot showing the pooled rate of permanent recurrent laryngeal nerve injury (RLNI) stratified by patient- and surgery-related characteristics. For each subgroup, the pooled rate is presented as a proportion (%) with corresponding 95% confidence intervals (CI), calculated using a random-effects model. The size of each marker reflects the weight of the subgroup, and horizontal lines indicate the 95% CI. The I² statistic represents the degree of heterogeneity within each subgroup, and τ² reflects between-study variance. The p-values reported in the first row of each category correspond to tests for subgroup differences (Q-test), indicating whether there is a statistically significant difference in pooled RLNI rates across subgroups within that category.

A summary of the pooled meta-analytic estimates of transient RLNI based on patients’ and surgical characteristics is provided in **Figure 3**. Surgical type was a significant effect modifier (P = .001). Secondary surgery (2% [1%-3%]) had the highest rate of permanent RLNI. Primary thyroidectomy, near-total, and sub-total procedures had 0% rates. Surgical method did not significantly affect permanent RLNI (P = .55). MIVAT was associated with a 1% rate; other techniques had 0%. Surgical approach was also not significant (P = .83); all approaches had 0% permanent RLNI rates. Surgeon type had no significant impact (P = .48). Consultants and residents both had 1% injury rates.

Hemostasis method was a significant modifier (P = .001). HemoClips were associated with the highest rate (3% [1%-6%]). LigaSure had a 0% rate; other techniques ranged around 1%. IONM was significant (P = .001), though the absolute RLNI rate remained 1% in both IONM and non-IONM groups. Drains were not significant (P = .48). RLNI rates were 1% regardless of drain use.

Routine preoperative laryngoscopy was not significant (P = .23), although higher detection of RLNI was observed with laryngoscopy (3% [0%-7%] vs 1% [1%-1%]). CND was a significant effect modifier (P = .001); however, no difference in absolute injury rates was observed (both 0%). Final pathology was significant (P = .001), although both benign and malignant groups had a 1% RLNI rate. Sex was not significant (P = .26); injury rates were 1% for both sexes.

Meta-regression (**Table 2**) showed that patient age (P = .043), HemoClips (P = .045), and secondary surgery (P < .001) were associated with increased RLNI risk. The model fit was excellent (R² = 100%; I² = 0%).

**Table 2:** Adjusted meta-regression analysis of determinants of permanent recurrent laryngeal nerve injury during thyroid surgery.

	Coefficient	SE	Z	P-value	5% CI	95% CI
Study design [reference group: RCT] – 126 studies						
Prospective Cohort	-0.0029	0.0205	-0.1400	0.8890	-0.0431	0.0373
Retrospective Cohort	-0.0105	0.0181	-0.5800	0.5610	-0.0459	0.0249
Surgical approach [reference group: endoscopic] – 21 studies						
Open	-0.0003	0.0019	-0.1700	0.8680	-0.0040	0.0034
MIVAT	-0.0002	0.0020	-0.1000	0.9240	-0.0042	0.0038
Robotic	0.0013	0.0053	0.2500	0.8040	-0.0091	0.0117
Surgical technique [reference group: transcervical] – 13 studies						
Transxillary	0.0124	0.0202	0.6100	0.5400	-0.0273	0.0521
Areolar	0.0000	(omitted)	0.0000	0.0000	0.0000	0.0000
**Follow-up** (per month) – 65 studies	0.0007	0.0007	1.0400	0.2960	-0.0006	0.0021
**Final Pathology** (Malignant vs. Not) – 56 studies	-0.0007	0.0010	-0.6600	0.5110	-0.0027	0.0014
**Final Pathology** (Benign vs. Not) – 56 studies	-0.0008	0.0010	-0.7600	0.4490	-0.0028	0.0012
**IONM** [Reference Group: None] – 127 studies	-0.0057	0.0065	-0.8800	0.3800	-0.0185	0.0070
**Age** (per year) – 42 studies	0.0003	0.0002	2.0300	0.0430	0.0000	0.0007
**M/F Ratio – 108 studies**	0.0000	0.0030	-0.0100	0.9930	-0.0059	0.0058
**Surgical Drain Use** [Reference Group: None] – 127 studies	-0.0008	0.0072	-0.1200	0.9080	-0.0149	0.0132
**Routine Preoperative Laryngoscopy** [Reference Group: None] – 127 studies	0.0001	0.0046	0.0200	0.9810	-0.0090	0.0092
**CND** [Reference Group: None] – 19 studies	0.0003	0.0002	1.0700	0.2840	-0.0002	0.0008
Homeostasis method [reference group: conventional ligation] – 127 studies						
Harmonic Scalpel	-0.0010	0.0021	-0.4600	0.6490	-0.0051	0.0032
LigaSure	-0.0010	0.0027	-0.3700	0.7150	-0.0063	0.0044
Electrocautery	0.0012	0.0016	0.7800	0.4330	-0.0019	0.0044
HemoClips	0.0457	0.0227	2.0100	0.0450	0.0011	0.0902
**Completion Thyroidectomy** [Reference Group: None] – 18 studies	0.0004	0.0014	0.3000	0.7640	-0.0023	0.0032
**Secondary/Revision Thyroidectomy** [Reference Group: Primary Thyroidectomy] – 127 studies	0.0001	0.0000	4.7800	0.0000	0.0001	0.0002
**Resident-Performed Surgery** [Reference Group: Consultant-Performed] – 16 studies	0.0000	(omitted)	0.0000	0.0000	0.0000	0.0000
**constant**	0.0056	0.0038	1.4700	0.1410	-0.0018	0.0130

Some covariates were excluded from this model either because of insufficient observations (i.e., surgery type) or omitted due to high multicollinearity, defined with variance inflation factor > 10. SE, standard error; CI, confidence interval; CND, central neck dissection; LND, lateral neck dissection; M/F, male/female ratio; IONM, intraoperative nerve monitoring; MIVAT, minimally-invasive video-assisted thyroidectomy; RCT, randomized controlled trial. The bold values indicate the most significant values within each category.

## Discussion

This meta-analysis demonstrates considerable variation in RLNI rates based on surgical type, approach, and technique, providing clinically actionable insights to guide thyroid surgery planning.

### Transient RLNI

Transient RLNI was most frequent following completion, secondary, and hemithyroidectomy, consistent with prior data (6, 35) showing elevated risk in reoperations due to scar tissue, altered anatomy, and reduced RLN visibility (36, 37). The higher risk in hemithyroidectomy may reflect difficulty preserving the RLN in cases involving large or posterior nodules (38). Although tumor size and location are known risk factors, inconsistent reporting—only 21 studies included size, often with nonstandard units—prevented stratified analysis.

Approach significantly influenced risk. Transoral and transcervical techniques were associated with the highest RLNI rates, likely due to restricted exposure and limited instrument mobility near the RLN (39, 40). In contrast, the transaxillary approach showed the lowest risk, possibly reflecting enhanced visualization. However, patients undergoing remote-access or minimally invasive surgery often have more favorable disease profiles (41), whereas transcervical surgery is typically reserved for larger tumors or malignancy (42). This selection bias limits direct comparison of techniques, despite adjustment for factors such as malignancy and central neck dissection.

IONM use was not associated with reduced transient RLNI. While widely adopted, its effectiveness is likely user-dependent and influenced by variability in protocols, device application, and surgeon experience (26, 43–47). However, subgroup analysis did not demonstrate a statistically significant difference in RLNI rates based on surgery performer, and therefore, no definitive conclusions can be drawn regarding the role of technical expertise as a primary determinant of nerve preservation.

Routine preoperative laryngoscopy did not alter injury rates, supporting its role as a diagnostic tool rather than a preventive measure (48). Its utility lies in identifying baseline RLN dysfunction and guiding operative planning, particularly in malignancy or revision settings.

Drain use was associated with higher RLNI rates but likely reflects case complexity rather than causality. Drains are typically placed in patients undergoing extensive dissection, reoperation, or treatment for large or invasive tumors—scenarios already associated with elevated RLNI risk (49, 50). This should not dissuade appropriate drain use when clinically indicated.

### Permanent RLNI

Permanent RLNI, though less frequent, has lasting functional consequences. Revision and completion thyroidectomies carried the highest risk, aligning with established evidence of increased technical difficulty in reoperative fields. Hemithyroidectomy, lobectomy, and near-total thyroidectomy were associated with lower rates.

Surgical method (open, endoscopic, robotic) did not significantly influence permanent RLNI, suggesting that surgeon skill and case selection may be more relevant than technique. Although newer methods offer cosmetic advantages and shorter recovery, their benefit in nerve preservation remains unproven and warrants further investigation as experience expands.

IONM also did not reduce permanent RLNI. This, combined with equivalent outcomes between consultants and residents (1), emphasizes that meticulous technique and structured training are critical regardless of adjunctive technologies.

### Determinants of RLNI

Meta-regression identified several independent determinants. MIVAT was associated with reduced transient RLNI compared with endoscopic surgery, likely due to smaller dissection zones and enhanced visualization (51). HemoClips were linked to increased permanent RLNI compared to conventional ligation, potentially due to mechanical compression or misplacement near the nerve (52).

Older age and drain use also were associated with higher RLNI risk. Age-related tissue fragility may increase vulnerability to manipulation (53), while drain use—as discussed—likely reflects surgical complexity (54).

The divergent significance patterns observed between transient and permanent RLNI for IONM, surgical approach, and surgical method warrant mechanistic and statistical consideration. Specifically, IONM did not significantly modify transient RLNI risk (p = 0.48) yet emerged as a significant determinant of permanent RLNI (p = 0.001), whereas surgical approach and method were significant modifiers of transient RLNI (p = 0.001 for both) but not of permanent RLNI (p = 0.83 and p = 0.55, respectively). These contrasting patterns likely reflect the distinct pathophysiological substrates of each injury type. Transient RLNI is predominantly attributable to reversible neuropraxic insults—including traction injury, thermal diffusion, and transient ischemia—whose severity is directly proportional to the degree of perineural dissection and instrument proximity to the nerve. Variables that define the surgical corridor and exposure geometry, namely operative approach and technique, therefore exert the greatest influence on transient injury rates. Permanent RLNI, by contrast, implies axonotmetic or neurotmetic injury with limited capacity for functional recovery. In this context, the real-time electrophysiological feedback afforded by IONM may confer a meaningful advantage by enabling intraoperative recognition of evolving signal loss and allowing timely modification of surgical strategy before irreversible damage is sustained—a protective mechanism of lesser relevance for the transient, self-resolving injuries that IONM cannot prospectively prevent. This interpretation is consistent with prior evidence suggesting that the clinical benefit of IONM may be most pronounced in high-stakes scenarios where injury would otherwise be permanent (43–47). An additional statistical consideration pertains to the near-zero absolute rates of permanent RLNI across virtually all subgroups in the present analysis. Under such conditions of extreme floor-level event rates, even trivially small absolute differences between subgroups can achieve statistical significance within a large pooled dataset, without necessarily reflecting clinically meaningful variation. Conversely, when absolute rates approach zero uniformly across categories—as observed for surgical approach and method in **Figure 3**—the discriminative capacity of subgroup analysis is inherently limited, irrespective of sample size. These observations collectively highlight the importance of interpreting subgroup p-values in conjunction with absolute effect magnitudes, and reinforce the need to treat transient and permanent RLNI as mechanistically and analytically distinct endpoints in future investigations.

### Clinical implications

These results inform perioperative decision-making. Surgeons should anticipate higher RLNI risk in reoperations, malignancy, and CND, and consider tailored strategies including selective IONM and preoperative laryngoscopy. MIVAT may offer safety advantages in appropriately selected cases, though broader comparisons across techniques must account for baseline differences. Avoidance of unnecessary neck dissection and refinement of hemostatic technique near the RLN may further mitigate injury risk.

### Limitations

This review is limited by heterogeneity in patient populations, surgical strategies, and outcome reporting. Most included studies were observational, introducing potential bias. Key tumor-specific variables (e.g., size, invasiveness, RLN proximity) were incompletely reported, limiting subgroup analysis. Similarly, limited data on surgeon experience, approach-by-type combinations, and IONM specifics (e.g., stimulation thresholds) constrained interpretation.

Preoperative laryngoscopy was inconsistently documented, making it difficult to differentiate pre-existing from surgery-related RLNI—particularly in advanced malignancies. Additionally, definitions of transient and permanent RLNI varied: 100 studies used a 6-month cutoff, 19 used 12 months, 3 used 3 months, and 77 did not define a threshold. Although laryngoscopy was universally used to confirm RLNI, follow-up durations were inconsistent, potentially affecting comparability.

## Conclusions

RLNI risk is significantly influenced by surgical type, approach, and intraoperative decisions. Reoperations and extensive procedures carry the highest risk. While IONM may be useful in select contexts, technical skill and case selection remain paramount. Standardized definitions, improved reporting of tumor characteristics, and further study of technology-specific factors are needed to optimize nerve preservation and surgical outcomes.

## Data Availability

The original contributions presented in the study are included in the article/**Supplementary Material**. Further inquiries can be directed to the corresponding author.

## Supplementary material

Supplementary Figure 1 The level of risk of bias of included randomized controlled trials using the revised Cochrane risk of bias tool (RoB-2).
