# Supplementary figures and images for: Prevalence and determinants of recurrent laryngeal nerve injury after thyroidectomy: a Systematic Review and meta-analysis
Source: Front Endocrinol (Lausanne). 2026 Apr 29;17:1764332. doi: 10.3389/fendo.2026.1764332 (PMC13167572; doi:10.3389/fendo.2026.1764332)

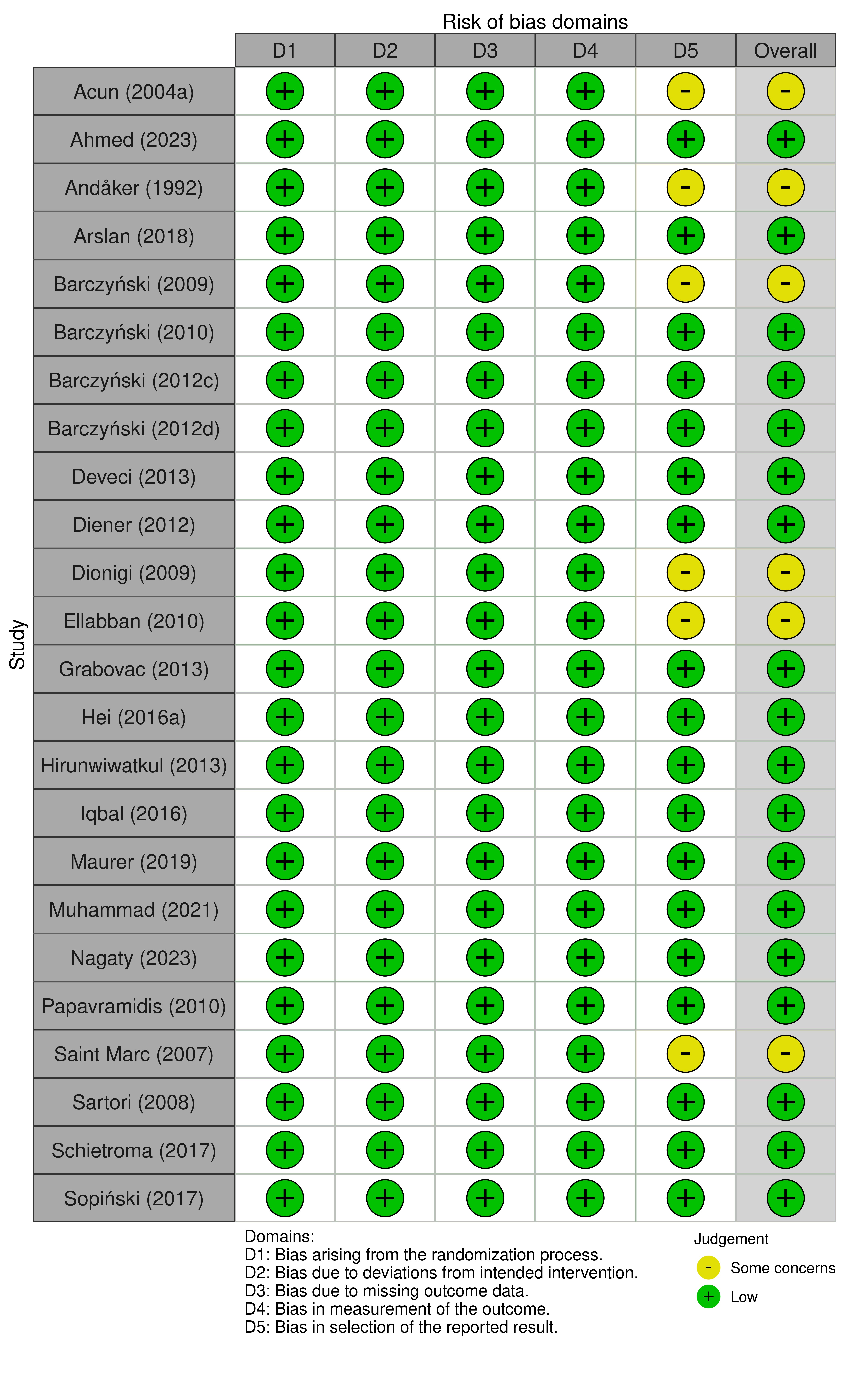

Supplement: Supplementary Figure 1 — The level of risk of bias of included randomized controlled trials using the revised Cochrane risk of bias tool (RoB-2). [file Image1.jpeg]
