# Supplementary material for: Prevalence and determinants of recurrent laryngeal nerve injury after thyroidectomy: a Systematic Review and meta-analysis
Source: Front Endocrinol (Lausanne). 2026 Apr 29;17:1764332. doi: 10.3389/fendo.2026.1764332 (PMC13167572; doi:10.3389/fendo.2026.1764332)
Supplement: Supplementary file 2 [file Table1.docx]

**Supplemental Table 1:** The search query employed in the literature search of this meta-analysis study is outlined below

| Database | No | Search Query | Results |
| --- | --- | --- | --- |
| PubMed [Date of Search 7/19/2024] | | | |
|  | #1 | Dysphonia[tiab] OR "Dysphonia"[Mesh] | 7014 |
|  | #2 | “recurrent laryngeal nerve”[tiab] OR "Recurrent Laryngeal Nerve"[Mesh] | 6780 |
|  | #3 | Injur*[tiab] OR damage[tiab] OR palsy[tiab] OR palsies[tiab] OR paralysis[tiab] OR impairment[tiab] OR paresis[tiab] | 2088759 |
|  | #4 | #2 AND #3 | 4578 |
|  | #5 | #4 OR #1 | 11365 |
|  | #6 | Thyroidectom*[tiab] OR “Thyroidectomy"[Mesh] | 36116 |
|  | #7 | #5 AND #6 | 2147 |
|  | #8 | Filters applied: Last 20 years + Humans only | 1418 |
| Scopus [Date of Search 7/19/2024] | | | |
|  | #1 | TITLE-ABS-KEY (Dysphonia) | 15266 |
|  | #2 | TITLE-ABS-KEY (“recurrent laryngeal nerve”) | 11677 |
|  | #3 | TITLE-ABS-KEY (Injur*) OR TITLE-ABS-KEY (damage) OR TITLE-ABS-KEY (palsy) OR TITLE-ABS-KEY (palsies) OR TITLE-ABS-KEY (paralysis) OR TITLE-ABS-KEY (impairment) OR TITLE-ABS-KEY (paresis) | 4318505 |
|  | #4 | #2 AND #3 | 8931 |
|  | #5 | #4 OR #1 | 12520 |
|  | #6 | TITLE-ABS-KEY (Thyroidectom*) | 51401 |
|  | #7 | #5 AND #6 | 3765 |
|  | #8 | Filters applied: Last 20 years + Humans only | 2143 |
| Web of Science [Date of Search 7/19/2024] | | | |
|  | #1 | AB=Dysphonia | 4948 |
|  | #2 | AB=“recurrent laryngeal nerve” | 4611 |
|  | #3 | AB=Injur* OR AB=damage OR AB=palsy OR AB=palsies OR AB=paralysis OR AB=impairment OR AB=paresis | 2460423 |
|  | #4 | #2 AND #3 | 3348 |
|  | #5 | #4 OR #1 | 8167 |
|  | #6 | AB=Thyroidectom* | 20359 |
|  | #7 | #5 AND #6 | 1386 |
|  | #8 | Filters applied: Last 20 years + Humans only | 1000 |
| CENTRAL [Date of Search 7/19/2024] | | | |
|  | #1 | Dysphonia | 1044 |
|  | #2 | “recurrent laryngeal nerve” | 441 |
|  | #3 | Injur* OR damage OR palsy OR palsies OR paralysis OR impairment OR paresis | 158451 |
|  | #4 | #2 AND #3 | 356 |
|  | #5 | #4 OR #1 | 1382 |
|  | #6 | Thyroidectom* | 2237 |
|  | #7 | #5 AND #6 | 213 |
|  | #8 | Filters applied: Last 20 years + Humans only + Trials only | 205 |
| Google Scholar [Date of Search 7/19/2024] | | | |
|  | With all of the words | thyroidectomy | - |
|  | With the exact phrase | recurrent laryngeal nerve | - |
|  | With at least one of the words | Injury damage palsy palsies paralysis impairment dysphonia paresis | - |
|  | Total | As per guidelines, only the first 200 records were retrieved | 200 |

AB: Abstract; KEY: keyword; tiab: title/abstract.
