# Supplementary material for: Prevalence and determinants of recurrent laryngeal nerve injury after thyroidectomy: a Systematic Review and meta-analysis
Source: Front Endocrinol (Lausanne). 2026 Apr 29;17:1764332. doi: 10.3389/fendo.2026.1764332 (PMC13167572; doi:10.3389/fendo.2026.1764332)
Supplement: Supplementary file 3 [file Table2.docx]

**Supplemental Table 2.** A list of finally selected articles in this review

| ID | Authors | Title |
| --- | --- | --- |
| 11 | Abdelhamid (2021) | Intraoperative nerve monitoring in thyroid surgery: analysis of United Kingdom registry of endocrine and thyroid surgery database |
| 21 | Acun (2004a) | A randomized prospective study of complications between general surgery residents and attending surgeons in near-total thyroidectomies |
| 23 | Acun (2005) | Importance of identifying the course of the recurrent laryngeal nerve in total and near-total thyroid lobectomies |
| 27 | Afolabi (2016) | A fifteen year experience of total thyroidectomy for the management of simple multinodular goitres in a low medium income country |
| 36 | Ahmed (2023) | Thyroidectomy With or Without Nerve Identification: A Personal Experience and Technique |
| 44 | Akici (2020) | Should intraoperative nerve monitoring be used routinely in primary thyroid surgeries? |
| 47 | Akkari (2014) | Thyroid surgery in children and adolescents: a series of 65 cases |
| 63 | Alesina (2012) | Intraoperative neuromonitoring does not reduce the incidence of recurrent laryngeal nerve palsy in thyroid reoperations: results of a retrospective comparative analysis |
| 65 | Al-Hakami (2019) | Surgical Complications After Thyroid Surgery: A 10-Year Experience at Jeddah, Saudi Arabia |
| 66 | Alhan (2015) | Total Thyroidectomy for Management of Benign Multinodular Goitre in an Endemic Region: Review of 620 Case |
| 68 | Alharbi (2018) | Experience of thyroid surgery at tertiary referral centers in Jazan Hospitals, Saudi Arabia |
| 71 | Ali (2012) | Outcome of surgery for toxic goitres in Maiduguri: a single teaching hospital's perspective |
| 69 | Ali (2019) | Outcomes After Urgent Thyroidectomy Following Rapid Control of Thyrotoxicosis in Graves' Disease are Similar to Those After Elective Surgery in Well-Controlled Disease |
| 70 | Ali (2022) | Frequency of Recurrent Laryngeal Nerve Injury in Thyroid Surgery For Benign Thyroid Disease |
| 78 | Almosallam (2020) | Thyroid surgery in 103 children in a single institution from 2000-2014 |
| 83 | Alqahtani (2023) | Recurrent laryngeal nerve injury after thyroidectomy: A national study from Saudi Arabia |
| 89 | AlSaiegh (2020) | Correlation between types of thyroid surgery, goitre pathology, and recurrent laryngeal nerve injury-retrospective cohort study |
| 101 | Ambe (2014) | Prolonged duration of surgery is not a risk factor for postoperative complications in patients undergoing total thyroidectomy: a single center experience in 305 patients |
| 106 | Amer (2022) | Immediate Recurrent Laryngeal Nerve Repair During Thyroidectomy |
| 112 | Andåker (1992) | Surgery for hyperthyroidism: hemithyroidectomy plus contralateral resection or bilateral resection? A prospective randomized study of postoperative complications and long-term results |
| 121 | Anuwong (2018) | Safety and Outcomes of the Transoral Endoscopic Thyroidectomy Vestibular Approach |
| 126 | Arikan (2023) | Transoral Thyroidectomy: Initial Results of the European TOETVA Study Group |
| 129 | Arslan (2018) | Is the superior laryngeal nerve really safe when using harmonic focus in total thyroidectomy? A prospective randomized study |
| 139 | Ay (2013) | SHOULD WE PREFER TOTAL TO NEAR-TOTAL THYROIDECTOMY IN DIFFUSE THYROID DISORDERS? |
| 146 | Aygun (2022) | Clinical and anatomical factors affecting recurrent laryngeal nerve paralysis during thyroidectomy via intraoperative nerve monitorization |
| 175 | Ban (2014) | Surgical complications after robotic thyroidectomy for thyroid carcinoma: a single center experience with 3,000 patients |
| 183 | Barczyński (2009) | Randomized clinical trial of visualization versus neuromonitoring of recurrent laryngeal nerves during thyroidectomy |
| 185 | Barczyński (2010) | Five-year follow-up of a randomized clinical trial of total thyroidectomy versus Dunhill operation versus bilateral subtotal thyroidectomy for multinodular nontoxic goiter |
| 181 | Barczynski (2012b) | Minimally invasive video-assisted thyroidectomy: seven-year experience with 240 cases |
| 187 | Barczyński (2012c) | Randomized clinical trial of bilateral subtotal thyroidectomy versus total thyroidectomy for Graves' disease with a 5-year follow-up |
| 188 | Barczyński (2012d) | Randomized controlled trial of visualization versus neuromonitoring of the external branch of the superior laryngeal nerve during thyroidectomy |
| 190 | Barczyński (2014) | Intraoperative nerve monitoring can reduce prevalence of recurrent laryngeal nerve injury in thyroid reoperations: results of a retrospective cohort study |
| 203 | Baud (2022) | Impact of Lymph Node Dissection on Postoperative Complications of Total Thyroidectomy in Patients with Thyroid Carcinoma |
| 206 | Bawa (2021) | Post-thyroidectomy complications in southwestern Saudi Arabia: a retrospective study of a 6-year period |
| 219 | Benkhadoura (2017) | Recurrent laryngeal nerve injury and hypoparathyroidism rates in reoperative thyroid surgery |
| 223 | Bergenfelz (2008) | Complications to thyroid surgery: Results as reported in a database from a multicenter audit comprising 3,660 patients |
| 224 | Bergenfelz (2016) | Risk of recurrent laryngeal nerve palsy in patients undergoing thyroidectomy with and without intraoperative nerve monitoring |
| 226 | Bertelli (2021) | Trans Oral Endoscopic Thyroidectomy Vestibular Approach (TOETVA) in Brazil: Safety and complications during learning curve |
| 241 | Bihain (2021) | What is the impact of continuous neuromonitoring on the incidence of injury to the recurrent laryngeal nerve during total thyroidectomy? |
| 279 | Bryk (2024) | The effect of intraoperative neuromonitoring on damage to the laryngeal nerves in patients undergoing total thyroidectomy |
| 281 | Bukarica (2022) | Thyroid Surgery in Children: A 5-Year Retrospective Study at a Single Paediatric Surgical Center and Systematic Review |
| 304 | Calò (2014a) | Role of intraoperative neuromonitoring of recurrent laryngeal nerves in the outcomes of surgery for thyroid cancer |
| 310 | Calò (2014b) | Identification alone versus intraoperative neuromonitoring of the recurrent laryngeal nerve during thyroid surgery: experience of 2034 consecutive patients |
| 311 | Calò (2014c) | Total thyroidectomy without prophylactic central neck dissection in clinically node-negative papillary thyroid cancer: Is it an adequate treatment? |
| 345 | Čelakovský (2011) | Risk factors for recurrent laryngeal nerve palsy after thyroidectomy |
| 346 | Celik (2011) | The Factors Related with Postoperative Complications in Benign Nodular Thyroid Surgery |
| 357 | Chan (2006) | The role of intraoperative neuromonitoring of recurrent laryngeal nerve during thyroidectomy: a comparative study on 1000 nerves at risk |
| 373 | Chaudhary (2007) | Recurrent laryngeal nerve injury: an experience with 310 thyroidectomies |
| 404 | Chen (2021) | Transoral robotic thyroidectomy versus transoral endoscopic thyroidectomy: a propensity-score-matched analysis of surgical outcomes |
| 382 | Chen (2022a) | Effect of gasless endoscopic thyroidectomy through an axillary approach on the recurrent laryngeal nerve injury in patients with thyroid cancer |
| 410 | Chen (2022b) | Safety and feasibility of the transoral endoscopic thyroidectomy vestibular approach with neuroprotection techniques for papillary thyroid carcinoma |
| 416 | Chereau (2024) | Impact of prophylactic central lymph node dissection on the complications and recurrence rates in papillary thyroid carcinoma-An AFCE (French-speaking Association of Endocrine Surgery) multicentre study based on the EUROCRINE® national data |
| 425 | Chiang (2004) | Risk of vocal palsy after thyroidectomy with identification of the recurrent laryngeal nerve |
| 433 | Chiang (2011) | Does extensive dissection of recurrent laryngeal nerve during thyroid operation increase the risk of nerve injury? Evidence from the application of intraoperative neuromonitoring |
| 448 | Chohan (2019) | Comparison of total and subtotal thyroidectomy in multinodular goiter |
| 471 | Chuang (2013) | Protective effect of intraoperative nerve monitoring against recurrent laryngeal nerve injury during re-exploration of the thyroid |
| 541 | De Palma (2016) | Post-thyroidectomy complications. The role of the device: bipolar vs ultrasonic device: Collection of data from 1,846 consecutive patients undergoing thyroidectomy |
| 548 | Dedhia (2020) | Outcomes after completion thyroidectomy versus total thyroidectomy for differentiated thyroid cancer: A single-center experience |
| 571 | Deveci (2013) | Is the use of a drain for thyroid surgery realistic? A prospective randomized interventional study |
| 583 | Diener (2012) | Vascular clips versus ligatures in thyroid surgery--results of a multicenter randomized controlled trial (CLIVIT Trial) |
| 600 | Dionigi (2009) | Neuromonitoring and video-assisted thyroidectomy: a prospective, randomized case-control evaluation |
| 633 | D'Orazi (2019) | May predictors of difficulty in thyroid surgery increase the incidence of complications? Prospective study with the proposal of a preoperative score |
| 642 | Dralle (2004) | Risk factors of paralysis and functional outcome after recurrent laryngeal nerve monitoring in thyroid surgery |
| 678 | Efremidou (2009) | The efficacy and safety of total thyroidectomy in the management of benign thyroid disease: a review of 932 cases |
| 685 | El-Labban (2009) | Minimally invasive video-assisted thyroidectomy versus conventional thyroidectomy: a single-blinded, randomized controlled clinical trial |
| 686 | El-labban (2010) | Comparison of minimally invasive video-assisted thyroidectomy and conventional thyroidectomy: a single-blinded, randomized controlled clinical trial |
| 696 | Emre (2008) | Complications of total thyroidectomy performed by surgical residents versus specialist surgeons |
| 697 | Emre (2016) | Efficacy of Intraoperative Single Dose Methylprednisolone on Recurrent Laryngeal Nerve Function After Thyroidectomy |
| 703 | Enomoto (2014) | Recurrent laryngeal nerve palsy during surgery for benign thyroid diseases: risk factors and outcome analysis |
| 707 | Erbil (2007) | Predictive factors for recurrent laryngeal nerve palsy and hypoparathyroidism after thyroid surgery |
| 710 | Erçetin (2019) | Is intraoperative nerve monitoring useful for surgical training in thyroid surgery? |
| 726 | Farizon (2017) | Intraoperative monitoring of the recurrent laryngeal nerve by vagal nerve stimulation in thyroid surgery |
| 730 | Fassari (2024) | Impact of Intermittent Intraoperative Neuromonitoring (IONM) on the Learning Curve for Total Thyroidectomy by Residents in General Surgery |
| 732 | Fei (2022) | Intraoperative neuromonitoring of the recurrent laryngeal nerve is indispensable during complete endoscopic radical resection of thyroid cancer: A retrospective study |
| 749 | Fiorelli (2021) | Anatomical and developmental aspects of iatrogenic injury to the right recurrent laryngeal nerve in surgical resections of substernal goiter |
| 758 | Formanez (2016) | Vocal fold paralysis with intraoperative recurrent laryngeal nerve identification versus non-identification of recurrent laryngeal nerve in total thyroidectomy: a retrospective cohort study |
| 764 | Frattini (2010) | Intraoperative neuromonitoring for thyroid malignancy surgery: technical notes and results from a retrospective series |
| 794 | Gao (2015) | Bilateral areolar approach endoscopic thyroidectomy for low-risk papillary thyroid carcinoma: a review of 137 cases [corrected] |
| 824 | Giulea (2015) | EVALUATION OF RECURRENTIAL COMPLICATIONS AFTER TOTAL THYROIDECTOMY |
| 823 | Giulea (2019) | Total thyroidectomy for malignancy - is central neck dissection a risk factor for recurrent nerve injury and postoperative hypocalcemia? A tertiary center experience in romania |
| 829 | Godballe (2014) | Risk factors for recurrent nerve palsy after thyroid surgery: a national study of patients treated at Danish departments of ENT Head and Neck Surgery |
| 849 | Grabovac (2013) | Comparison of recurrent nerve injuries during thyroidectomy performed by conventional or harmonic scalpel |
| 858 | Gremillion (2012) | Intraoperative Recurrent Laryngeal Nerve Monitoring in Thyroid Surgery: Is It Worth the Cost? |
| 875 | Gunn (2020) | Recurrent laryngeal nerve injury after thyroid surgery: an analysis of 11,370 patients |
| 884 | Gür (2019) | Intraoperative nerve monitoring during thyroidectomy: evaluation of signal loss, prognostic value and surgical strategy |
| 896 | Gurrado (2016) | Can Total Thyroidectomy Be Safely Performed by Residents?: a Comparative Retrospective Multicenter Study |
| 901 | Gutierrez-Alvarez (2023) | Advantages of Intraoperative Neuromonitoring Over Direct Visualization of the Recurrent Laryngeal Nerve During Thyroidectomy |
| 905 | Haddadin (2023) | Comparison of recurrent laryngeal nerve insult incidence post thyroidectomy for benign and malignant lesions |
| 917 | Hamilton (2019) | Continuous intraoperative nerve monitoring in thyroidectomy using automatic periodic stimulation in 256 at-risk nerves |
| 919 | Hammad (2016) | A Prospective Study Comparing the Efficacy and Surgical Outcomes of Harmonic Focus Scalpel Versus LigaSure Small Jaw in Thyroid and Parathyroid Surgery |
| 923 | Hardman (2015) | Re-operative thyroid surgery: a 20-year prospective cohort study at a tertiary referral centre |
| 929 | Hasin (2020) | Incidence of early complications in laparoscopic total thyroidectomy vs open thyroidectomy using breast approach in simple Multinodular goiter |
| 942 | Hei (2016a) | Intermittent intraoperative nerve monitoring in thyroid reoperations: preliminary results of a randomized, single-surgeon study |
| 943 | Hei (2016b) | Intermittent Intraoperative Neural Monitoring Technology in Minimally Invasive Video-Assisted Thyroidectomy: a Preliminary Study |
| 961 | Hindosh (2011) | The incidence of recurrent laryngeal nerve injury during thyroid surgery |
| 964 | Hirsch (2014) | Total versus hemithyroidectomy for small unilateral papillary thyroid carcinoma |
| 966 | Hirunwiwatkul (2013) | A multicenter, randomized, controlled clinical trial of LigaSure small jaw vessel sealing system versus conventional technique in thyroidectomy |
| 969 | Hoff (2024) | Thyroidectomy for Euthyroid Patients with Hashimoto Disease and Persistent Symptoms: An Observational, Postrandomization Study |
| 985 | Hu (2016) | Total thyroidectomy as primary surgical management for thyroid disease: Surgical therapy experience from 5559 thyroidectomies in a less-developed region |
| 990 | Huang (2015) | The preoperative evaluation prevent the postoperative complications of thyroidectomy |
| 1030 | Idris (2013) | Incidence of recurrent laryngeal nerves injury during thyroid surgery |
| 1037 | Iqbal (2016) | Damage to Recurrent Laryngeal Nerve (RLN) with and without Exposure in Thyroidectomy |
| 1062 | Jawad (2018) | Recurrent Laryngeal Nerve Injury With Versus Without Nerve Identification In Different Thyroidectomy Procedures |
| 1085 | Joliat (2017) | Recurrent laryngeal nerve injury after thyroid and parathyroid surgery: Incidence and postoperative evolution assessment |
| 1091 | Jonas (2006) | [Intraoperative neuromonitoring of the recurrent laryngeal nerve - results and learning curve] |
| 1101 | Kai (2017) | Intraoperative nerve monitoring reduces recurrent laryngeal nerve injury in geriatric patients undergoing thyroid surgery |
| 1135 | Karpathiotakis (2022) | Intraoperative Neuromonitoring and Optical Magnification in the Prevention of Recurrent Laryngeal Nerve Injuries during Total Thyroidectomy |
| 1161 | Khan (2022) | COMPARISON OF RECURRENT LARYNGEAL NERVE (RLN) PALSY WITH AND WITHOUT INTRAOPERATIVE NERVE IDENTIFICATION DURING THYROIDECTOMY – A CROSS-SECTIONAL STUDY FROM A TERTIARY LEVEL HOSPITAL IN PESHAWAR |
| 1180 | Kim (2021) | Intraoperative nerve monitoring is associated with a lower risk of recurrent laryngeal nerve injury: A national analysis of 17,610 patients |
| 1246 | Kumar (2019) | Risk Factors for Injury to Recurrent Laryngeal Nerve in Thyroid Surgeries-A Tertiary Care Centre Experience |
| 1261 | Kuryga (2021) | Training in intraoperative neuromonitoring of recurrent laryngeal nerves reduces the risk of their injury during thyroid surgery |
| 1293 | Landerholm (2014) | Incidence and risk factors for injuries to the recurrent laryngeal nerve during neck surgery in the moderate-volume setting |
| 1351 | Lenay-Pinon (2021) | The circumstances in which recurrent laryngeal nerve palsy occurs after surgery for benign thyroid disease: a retrospective study of 1026 patients |
| 1356 | Leow (2020) | Comparison of Outcomes of Intra-operative Neuromonitoring of Recurrent Laryngeal Nerve Versus Visualisation Alone during Thyroidectomies: A Singapore Experience |
| 1410 | Ling (2020) | Role of intraoperative neuromonitoring of recurrent laryngeal nerve in thyroid and parathyroid surgery |
| 1420 | Liu (2020) | Mechanisms of recurrent laryngeal nerve injury near the nerve entry point during thyroid surgery: a retrospective cohort study |
| 1421 | Liu (2021) | Recurrent laryngeal nerve injury near the nerve entry point in Total endoscopic thyroidectomy: a retrospective cohort study |
| 1494 | Machens (2018) | Long-term outcome of prophylactic thyroidectomy in children carrying RET germline mutations |
| 1500 | Maeda (2006) | Video-assisted subtotal or near-total thyroidectomy for Graves' disease |
| 1506 | Mahoney (2021) | Predictors and consequences of recurrent laryngeal nerve injury during open thyroidectomy: An American College of Surgeons National Surgical Quality Improvement Project database analysis |
| 1513 | Maksimoski (2022) | Outcomes in Pediatric Thyroidectomy: Results From a Multinational, Multi-institutional Database |
| 1527 | Marin Arteaga (2018) | Modification of the Surgical Strategy for the Dissection of the Recurrent Laryngeal Nerve Using Continuous Intraoperative Nerve Monitoring |
| 1544 | Maurer (2019) | Total Versus Near-total Thyroidectomy in Graves Disease: results of the Randomized Controlled Multicenter TONIG-trial |
| 1546 | Maurer (2020) | Short-Term Outcomes of Surgery for Graves' Disease in Germany |
| 1574 | Messenbaeck (2018) | Minimally invasive endoscopic thyroid surgery using a collar access: experience in 246 cases with the CEViTS technique |
| 1594 | Mirallié (2018) | Does intraoperative neuromonitoring of recurrent nerves have an impact on the postoperative palsy rate? Results of a prospective multicenter study |
| 1599 | Mismar (2024) | Complications after Thyroidectomy; a Comparison Among Seven Different Surgical Procedures and the Impact of Central Compartment Lymph Nodes Sampling: a Cohort Study |
| 1607 | Mizuno (2019) | Recurrent laryngeal nerve paralysis after thyroid cancer surgery and intraoperative nerve monitoring |
| 1610 | Mobayen (2015) | Comparison of the results of total thyroidectomy and Dunhill operation in surgical treatment of multinodular goiter |
| 1617 | Mohammad (2022) | Recurrent laryngeal nerve paralysis following thyroidectomy: analysis of factors affecting nerve recovery |
| 1625 | Molinari (2015) | Thyroid surgery performed on an overnight basis: a 17 years of experience |
| 1636 | Moreira (2020) | Investigation of recurrent laryngeal palsy rates for potential associations during thyroidectomy |
| 1647 | Muhammad (2021) | Intraoperative Nerve Monitoring Improves Junior Surgeon Detection Rate of Recurrent Laryngeal Nerve |
| 1651 | Mulita (2022) | Thyroidectomy for the Management of Differentiated Thyroid Carcinoma and their Outcome on Early Postoperative Complications: A 6-year Single-Centre Retrospective Study |
| 1669 | Nagaoka (2022) | Learning Curve for Endoscopic Thyroidectomy Using Video-Assisted Neck Surgery: Retrospective Analysis of a Surgeon's Experience with 100 Patients |
| 1670 | Nagaty (2023) | An assessment of the role of surgical loupe technique in prevention of postthyroidectomy complications: a comparative prospective study |
| 1691 | Nayyar (2020) | Risk factors predisposing for recurrent laryngeal nerve palsy following thyroid malignancy surgery: experience from a tertiary oncology centre |
| 1733 | Ngo (2023) | Transoral endoscopic thyroidectomy vestibular approach as a novel technique for pediatric populations: Results from a single surgeon |
| 1737 | Nguyen (2021) | Comparison of Transoral Thyroidectomy Vestibular Approach and Unilateral Axillobreast Approach for Endoscopic Thyroidectomy: a Prospective Cohort Study |
| 1785 | Ozbas (2005) | Comparison of the complications of subtotal, near total and total thyroidectomy in the surgical management of multinodular goitre |
| 1794 | Paek (2022) | A Comparison of the Bilateral Axillo-breast Approach (BABA) Robotic and Open Thyroidectomy for Papillary Thyroid Cancer after Propensity Score Matching |
| 1808 | Palmer (2005) | Papillary thyroid carcinoma in children: risk factors and complications of disease recurrence |
| 1813 | Pantvaidya (2018) | Does the recurrent laryngeal nerve recover function after initial dysfunction in patients undergoing thyroidectomy? |
| 1818 | Papavramidis (2010) | UltraCision harmonic scalpel versus clamp-and-tie total thyroidectomy: a clinical trial |
| 1839 | Park (2019) | Comparison of functional outcomes after total thyroidectomy and completion thyroidectomy: Hypoparathyroidism and postoperative complications |
| 1856 | Pei (2021) | The value of intraoperative nerve monitoring against recurrent laryngeal nerve injury in thyroid reoperations |
| 1861 | Pelizzo (2014) | Complications in thyroid resurgery: a single institutional experience on 233 patients from a whole series of 4,752 homogeneously treated patients |
| 1868 | Pergel (2014) | A safety-based comparison of pure LigaSure use and LigaSure-tie technique in total thyroidectomy |
| 1869 | Périé (2013) | Value of recurrent laryngeal nerve monitoring in the operative strategy during total thyroidectomy and parathyroidectomy |
| 1886 | Piccoli (2019) | Evolution Strategies in Transaxillary Robotic Thyroidectomy: Considerations on the First 449 Cases Performed |
| 1888 | Pieracci (2007) | Substernal thyroidectomy is associated with increased morbidity and mortality as compared with conventional cervical thyroidectomy |
| 1915 | Porseyedi (2012) | Comparison of the Frequency of Recurrent Laryngeal Nerve Injury with and without Exploration of the Nerve in Thyroidectomy |
| 1931 | Prokopakis (2013) | Intraoperative recurrent laryngeal nerve monitoring in revision thyroidectomy |
| 1949 | Qu (2021) | Clinical analysis of total endoscopic thyroidectomy via breast areola approach in early differentiated thyroid cancer |
| 1954 | Rafferty (2007) | Completion thyroidectomy versus total thyroidectomy: is there a difference in complication rates? An analysis of 350 patients |
| 1982 | Rasool (2020) | Total versus subtotal thyroidectomy for the management of benign multinodular goiter at dhq teaching hospital gujranwala |
| 1984 | Raval (2009) | Total thyroidectomy for benign disease in the pediatric patient--feasible and safe |
| 1988 | Razavi (2018) | Early outcomes in transoral vestibular thyroidectomy: Robotic versus endoscopic techniques |
| 1999 | Riju (2019) | Completion Thyroidectomy in Differentiated Thyroid Malignancy—A Prospective Analysis |
| 2003 | Ríos-Zambudio (2004) | Prospective study of postoperative complications after total thyroidectomy for multinodular goiters by surgeons with experience in endocrine surgery |
| 2005 | Ritter (2021) | Role of intraoperative recurrent laryngeal nerve monitoring for pediatric thyroid surgery: Comparative analysis |
| 2008 | Robertson (2004) | Continuous laryngeal nerve integrity monitoring during thyroidectomy: does it reduce risk of injury? |
| 2021 | Rosato (2004) | Complications of Thyroid Surgery: Analysis of a Multicentric Study on 14,934 Patients Operated on in Italy over 5 Years |
| 2024 | Rossi (2022) | Postsurgical complications after robot-assisted transaxillary thyroidectomy: critical analysis of a large cohort of European patients |
| 2030 | Rudolph (2014) | The Morbidity of Reoperative Surgery for Recurrent Benign Nodular Goitre: Impact of Previous Unilateral Thyroid Lobectomy versus Subtotal Thyroidectomy |
| 2039 | Russell (2021) | Transoral Thyroidectomy: Safety and Outcomes of 200 Consecutive North American Cases |
| 2065 | Saint Marc (2007) | LigaSure vs clamp-and-tie technique to achieve hemostasis in total thyroidectomy for benign multinodular goiter: A prospective randomized study |
| 2068 | Sajid (2016) | Recurrent laryngeal nerve injury in total versus subtotal thyroidectomy |
| 2084 | Sanguinetti (2014) | Intraoperative recurrent laryngeal nerve monitoring in thyroid surgery Evaluation of its use in terms of "spending review" |
| 2088 | Santosh (2014) | Preoperative Preparation with Lugol's Iodine in Thyroidectomy of Euthyroid Patients-Is it Really Mandatory?-An Otorhinolaryngologist's View |
| 2092 | Sarkis (2017) | Bilateral recurrent laryngeal nerve injury in a specialized thyroid surgery unit: would routine intraoperative neuromonitoring alter outcomes? |
| 2095 | Sartori (2008) | Ligasure versus Ultracision in thyroid surgery: a prospective randomized study |
| 2110 | Schietroma (2017) | Thyroid Surgery: to Drain or Not to Drain, That Is the Problem - A Randomized Clinical Trial |
| 2113 | Schneider (2019) | Complete and incomplete recurrent laryngeal nerve injury after thyroid and parathyroid surgery: Characterizing paralysis and paresis |
| 2145 | Sena (2019) | Total thyroidectomy vs completion thyroidectomy for thyroid nodules with indeterminate cytology/follicular proliferation: a single-centre experience |
| 2165 | Shakir (2016) | Outcome of Total Thyroidectomy for Bilateral Multinodular Thyroid Disease in tertiary care hospital |
| 2180 | Shen (2013) | Routine exposure of recurrent laryngeal nerve in thyroid surgery can prevent nerve injury |
| 2200 | Shindo (2007) | Incidence of vocal cord paralysis with and without recurrent laryngeal nerve monitoring during thyroidectomy |
| 2242 | Sleptsov (2023) | Tension-free thyroidectomy (medial thyroidectomy)-a prospective study: surgical technique and results of 259 operations |
| 2252 | Snyder (2010) | Outpatient thyroidectomy is safe and reasonable: experience with more than 1,000 planned outpatient procedures |
| 2254 | Snyder (2013) | The long-term impact of routine intraoperative nerve monitoring during thyroid and parathyroid surgery |
| 2277 | Sopiński (2017) | Role of intraoperative neuromonitoring of the recurrent laryngeal nerves during thyroid reoperations of recurrent goiter |
| 2293 | Sreejayan (2017) | Study of complications of thyroidectomy with special reference to recurrent laryngeal nerve injury |
| 2304 | Stevens (2012) | The impact of recurrent laryngeal neuromonitoring on multi-dimensional voice outcomes following thyroid surgery |
| 2343 | Tabriz (2024) | Risk Factors for Recurrent Laryngeal Nerve Palsy in Thyroid Surgery: A Single Center Experience of 1147 Procedures with Intermittent Intraoperative Neuromonitoring |
| 2386 | Testini (2014) | Recurrent laryngeal nerve palsy and substernal goiter. An Italian multicenter study |
| 2432 | Tsuzuki (2019) | Thyroid lobe size predicts risk of postoperative temporary recurrent laryngeal nerve paralysis |
| 2477 | Vasileiadis (2016) | Association of intraoperative neuromonitoring with reduced recurrent laryngeal nerve injury in patients undergoing total thyroidectomy |
| 2482 | Velayutham (2022) | Importance of Intraoperative Neuromonitoring Parameters in Predicting Temporary Recurrent Laryngeal Nerve Palsy Following Thyroid Surgery for Malignancy |
| 2489 | Veyseller (2011) | Effect of recurrent laryngeal nerve identification technique in thyroidectomy on recurrent laryngeal nerve paralysis and hypoparathyroidism |
| 2504 | Vural (2021) | Identification of the recurrent laryngeal nerve during thyroidectomy can affect the complication rate |
| 2505 | Waheed (2017) | FREQUENCY OF RECURRENT LARYNGEAL INJURY IN THYROIDECTOMY SURGERY |
| 2587 | Wojtczak (2017) | Experience with intraoperative neuromonitoring of the recurrent laryngeal nerve improves surgical skills and outcomes of non-monitored thyroidectomy |
| 2607 | Wu (2017) | Recurrent laryngeal nerve injury with incomplete loss of electromyography signal during monitored thyroidectomy—evaluation and outcome |
| 2619 | Wu (2018) | Routine Intraoperative Neuromonitoring of the Recurrent Laryngeal Nerve to Facilitate Complete Resection and Ensure Safety in Thyroid Cancer Surgery |
| 2636 | Xu (2023) | Mechanisms of recurrent laryngeal nerve injury in endoscopic thyroidectomy for papillary thyroid carcinoma: A large data from C hina |
| 2708 | Yu (2020) | Early detection of recurrent laryngeal nerve damage using intraoperative nerve monitoring during thyroidectomy |
| 2711 | Yu (2021) | Hyperthermal liquid, spray, and smog may be potential risk factors for recurrent laryngeal nerve thermal injury during thyroid surgeries |
| 2727 | Yuksekdag (2019) | Recurrent laryngeal nerve injury in total thyroidectomy with intraoperative nerve monitoring and harmonic sealing instrument: A retrospective analysis and treatment results |
