# Supplementary material for: Prevalence and determinants of recurrent laryngeal nerve injury after thyroidectomy: a Systematic Review and meta-analysis
Source: Front Endocrinol (Lausanne). 2026 Apr 29;17:1764332. doi: 10.3389/fendo.2026.1764332 (PMC13167572; doi:10.3389/fendo.2026.1764332)
Supplement: Supplementary file 4 [file Table3.docx]

**Supplemental Table 3:** Baseline characteristics of included studies investigating the rate of transient and permanent RLN injury during thyroid surgery

| Author (YOP) | Country | Design | YOI | Sample | Age | | Gender | | FU (mo) | Neuromonitoring (IONM) | | | Revision Surgery (%) | Surgical Drains | | Routine Preop Laryngoscopy | LN Dissection (%) | CND (%) |
| --- | --- | --- | --- | --- | --- | --- | --- | --- | --- | --- | --- | --- | --- | --- | --- | --- | --- | --- |
|  |  |  |  |  | Mean | SD | M | F |  | Yes/No | Type | Detail |  | % | Time (hours) |  |  |  |
| Abdelhamid (2021) | UK | RC | 2008-2018 | 42341 | - | (16-90) | - | - | - | - | - | methylene blue | - | - | - | Yes | - | - |
| Acun (2004a) | Turkey | RCT | 2001-2003 | 152 | 43 | (24-77) | 39 | 113 | 12 | 0/152 | - | Eye (traced to cricoid) | - | - | - | No | - | - |
| Acun (2005) | Turkey | RC | - | 176 | 44 | (23-77) | 49 | 127 | - | 0/176 | - | Eye (traced to cricoid) | - | - | - | No | - | - |
| Afolabi (2016) | Nigeria | RC | 2001-2015 | 447 | 39 | (15-74) | 62 | 385 | 12 | - | - | - | - | 100 | 48-72 h | No | - | - |
| Ahmed (2023) | Iraq | RCT | 2018-2020 | 150 | 39 | - | 22 | 128 | - | 75/75 | - | - | - | - | - | No | - | - |
| Akici (2020) | Turkey | RC | 2012-2017 | 273 | 47 | 12 | 38 | 235 | - | 140/133 | - | - | - | - | - | No | - | - |
| Akkari (2014) | France | RC | 2004-2012 | 65 | 12.5 | 0.7 | 16 | 49 | - | 40/25 | - | Binocular magnifying glass | - | - | - | No | 7.7 | - |
| Alesina (2012) | Germany | RC | 1999-2011 | 246 | 55 | 12.5 | 37 | 209 | - | 89/157 | - | - | 100 | 57.6 | - | No | 1.2 | - |
| Al-Hakami (2019) | KSA | RC | 2008-2017 | 456 | 42.6 | (10-89) | 99 | 357 | 12 | 456/0 | - | - | 21.27 | - | - | No | - | - |
| Alhan (2015) | Turkey | RC | 2004-2012 | 620 | 48 | 14 | 109 | 511 | 6 | 0/620 | - | - | - | - | - | No | - | - |
| Alharbi (2018) | KSA | RC | 2011-2018 | 320 | 42.25 | 9.5 | 112 | 208 | - | - | 0/620 | - | - | - | - | No | - | - |
| Ali (2012) | Nigeria | PC | 2005-2010 | 78 | - | - | 11 | 67 | - | - | - | - | - | - | - | No | - | - |
| Ali (2019) | UK | RC | 2010-2016 | 247 | 39 | (31-51) | 15 | 232 | 6 | - | - | - | - | - | - | No | - | - |
| Ali (2022) | Pakistan | CS | 2015-2018 | 179 | 37.9 | 8.3 | 42 | 137 | - | - | - | - | - | - | - | No | - | - |
| Almosallam (2020) | KSA | RC | 2000-2014 | 103 | 13.2 | (2-18) | 23 | 80 | - | - | - | - | - | - | - | No | - | - |
| Alqahtani (2023) | KSA | RC | 2015-2021 | 432 | 41.2 | 19.1 | 76 | 361 | 0.75 | 0/432 | - | - | - | - | - | Yes | - | - |
| AlSaiegh (2020) | Iraq | RC | 2007-2018 | 705 | - | (11-80) | 90 | 615 | 37 | - | - | - | - | - | - | No | - | - |
| Ambe (2014) | Germany | RC | 2006-2012 | 305 | 54.3 | 14.25 | 78 | 227 | - | 305/0 | - | - | - | - | - | Yes | - | - |
| Amer (2022) | Egypt | RC | 2012-2020 | 813 | 54.3 | 17.5 | - | - | - | - | - | - | - | - | - | No | - | - |
| Andåker (1992) | Sweden | RCT | 1986-1987 | 50 | 44 | 12.5 | 7 | 43 | - | - | - | - | - | - | - | No | - | - |
| Anuwong (2018) | Thailand | RC | 2014-2016 | 425 | 35.3 | 12.1 | 36 | 389 | 12 | - | - | - | - | 100 | - | No | - | - |
| Arikan (2023) | Austria, Germany, Italy, and Turkey | RC | 2016-2022 | 391 | 40.8 | 13.4 | 47 | 344 | - | - | - | - | - | - | - | No | - | - |
| Arslan (2018) | Turkey | RCT | 2013-2015 | 206 | 45 | (21-75) | 36 | 170 | 1 | - | - | - | - | 18.44 | - | Yes | - | - |
| Ay (2013) | Turkey | RC | 2004-2009 | 417 | - | - | - | - | 6 | - | - | - | - | - | - | No | - | - |
| Aygun (2022) | Turkey | RC | 2016-2021 | 871 | 49.17 | 13.42 | 199 | 672 | - | 871/0 | - | - | 5.9 | - | - | No | - | 10.4 |
| Ban (2014) | Korea | RC | 2007-2013 | 3000 | 30.9 | 9.1 | 307 | 2693 | 6 | - | - | - | - | - | - | No | - | - |
| Barczyński (2009) | Poland | RCT | 2006-2007 | 1000 | 51.6 | 14.6 | 88 | 912 | 12 | 500/500 | - | Neurosign | - | - | - | No | - | - |
| Barczyński (2010) | Poland | RCT | 2000-2003 | 600 | 47.22 | 15.61 | 53 | 517 | - | 0/600 | - | - | - | - | - | No | - | - |
| Barczynski (2012b) | Poland | RC | 2004-2011 | 240 | 40.4 | (18-63) | 228 | 12 | 6 | - | - | - | - | - | - | No | - | - |
| Barczyński (2012c) | OVERLAPPING DATA | RCT | 2000-2004 | 191 | 45.9 | (43.1-48.9) | 21 | 170 | 12 | 0/191 | - | - | - | - | - | Yes | - | - |
| Barczyński (2012d) | Poland | RCT | 2009-2010 | 210 | 49.9 | 14.7 | 0 | 201 | 6 | 100/101 | - | NIM 3.0 system | - | - | - | No | - | - |
| Barczyński (2014) | Poland | RC | 1993-2012 | 854 | 54.3 | 13.4 | 687 | 167 | - | 306/548 | - | - | 100 | - | - | Yes | - | 9.25 |
| Baud (2022) | France | RC | 2000-2009 | 1547 | 49.1 | 14.5 | 354 | 1193 | 12 | - | - | - | - | - | - | No | - | - |
| Bawa (2021) | KSA | RC | 2013-2019 | 339 | 38 | (29-48) | 59 | 280 | - | 0/339 | - | - | - | - | - | Yes | - | - |
| Benkhadoura (2017) | Libya | RC | 2002-2014 | 73 | 37 | (19-80) | 7 | 66 | 6 | - | - | - | 100 | - | - | Yes | - | - |
| Bergenfelz (2008) | Sweden | RC | 2004-2006 | 3660 | - | - | - | - | 12 | - | - | - | - | - | - | No | - | - |
| Bergenfelz (2016) | Sweden | RC | 2009-2013 | 5252 | 49 | (38-63) | 1050 | 4202 | 6 | 3277/1975 | - | - | 9.6 | - | - | Yes | 15.6 | - |
| Bertelli (2021) | Brazil | RC | 2017 | 93 | - | - | 14 | 79 | - | 93/0 | - | Neurosoft | - | - | - | No | - | - |
| Bihain (2021) | France | PC | 2013-2019 | 603 | 52.8 | 15 | 137 | 466 | 0.06 | 367/236 | - | Medtronic 3.0 | - | - | - | No | 10.12 | - |
| Bryk (2024) | Poland | RC | - | 367 | 52.2 | (18-79) | 55 | 312 | - | 205/162 | - | - | - | - | - | Yes | - | - |
| Bukarica (2022) | Serbia | RC | 2017-2022 | 17 | 14.8 | - | 3 | 14 | - | - | - | - | - | - | - | No | - | 17.6 |
| Calò (2014a) | Italy | RC | 2007-2013 | 656 | - | - | - | - | - | 357/299 | - | Medtronic | - | - | - | Yes | - | - |
| Calò (2014b) | Italy | RC | 2007-2012 | 2034 | - | - | - | - | - | 1041/993 | - | Medtronic | - | - | - | Yes | - | - |
| Calò (2014c) | Italy | RC | 2002-2010 | 285 | 51.54 | 16.39 | 60 | 225 | - | - | - | - | - | - | - | Yes | - | 22.8 |
| Čelakovský (2011) | Czech | RC | 2001-2005 | 1224 | - | - | - | - | 0.25 | - | - | - | - | - | - | Yes | - | - |
| Celik (2011) | Turkey | RC | 2004-2008 | 332 | 44.87 | 11.57 | 38 | 294 | 6 | - | - | - | - | - | - | No | - | - |
| Chan (2006) | China | RC | 2002-2005 | 639 | 49 | (8-93) | 133 | 506 | - | 501/499 | - | Neurosign 100 | 13.5 | - | - | Yes | - | - |
| Chaudhary (2007) | Pakistan | PC | 2000-2005 | 310 | - | (11-60) | 9 | 301 | - | - | - | - | - | - | - | Yes | - | - |
| Chen (2021) | Taiwan | RC | 2017-2019 | 150 | 45 | (36-56) | 18 | 136 | 6 | - | - | - | 4.5 | No | - | No | 26 | - |
| Chen (2022a) | China | RC | 2019-2020 | 110 | 41.1 | 7.25 | 46 | 64 | - | 110/0 | - | - | - | 100 | - | No | - | - |
| Chen (2022b) | China | RC | 2016-2020 | 75 | 36.8 | 10.5 | 9 | 66 | - | - | - | - | - | 100 | 67.2 | No | - | 100 |
| Chereau (2024) | France | RC | 2015-2021 | 1905 | 49 | (39-60) | 359 | 1546 | - | - | - | - | - | - | - | No | - | 80.52 |
| Chiang (2004) | Taiwan | RC | 1986-2002 | 521 | 42 | (17-78) | 118 | 403 | - | 0/521 | - | - | 5.56 | - | - | No | - | - |
| Chiang (2011) | Taiwan | RC | 2006-2009 | 231 | - | - | - | - | - | 231/0 | - | Medtronic | - | - | - | Yes | - | - |
| Chohan (2019) | Pakistan | PC | 2018 | 50 | 35.57 | 10.04 | 9 | 51 | - | - | - | - | - | - | - | No | - | - |
| Chuang (2013) | Taiwan | RC | 2001-2010 | 71 | - | (22.8-85) | 12 | 59 | - | 56/15 | - | Medtronic | 100 | - | - | No | - | - |
| De Palma (2016) | Italy | RC | 2010-2011 | 1846 | 52 | - | 395 | 1451 | 0.25 | - | - | - | - | - | - | No | - | 13.49 |
| Dedhia (2020) | USA | RC | 2000-2018 | 1096 | 50.1 | 0.78 | 341 | 755 | 6 | 1096/0 | - | - | - | - | - | No | - | - |
| Deveci (2013) | Turkey | RCT | 2010-2012 | 400 | 46.8 | 12.9 | 47 | 353 | - | - | - | - | - | 200/200 | 24 | No | - | - |
| Diener (2012) | Germany | RCT | 2004 | 491 | 54.7 | 12.5 | 127 | 364 | - | - | - | - | - | 365/123 | - | No | - | - |
| Dionigi (2009) | Italy | RCT | 2004-2007 | 72 | 40.5 | (19-77) | 10 | 62 | 12 | - | - | Medtronic 2.0 | - | No | - | No | - | - |
| D'Orazi (2019) | Italy | PC | 2015-2017 | 164 | 54 | (21-79) | 29 | 135 | - | - | - | - | - | - | - | No | - | - |
| Dralle (2004) | Germany | RC | 1998-2001 | 16448 | - | - | - | - | - | 12166/17832 | - | Neurosign 100 | - | - | - | Yes | - | - |
| Efremidou (2009) | Greece | RC | 1985-2005 | 932 | 49 | (16-82) | 194 | 738 | 12 | - | - | - | - | 100 | 48 | No | - | - |
| El-Labban (2009) | Italy | RC | 1998-2000 | 67 | - | - | - | - | - | - | - | - | - | - | - | No | - | - |
| El-labban (2010) | Egypt | RCT | 2002-2007 | 76 | 41 | 18 | 21 | 55 | 3 | - | - | - | - | - | - | No | - | - |
| Emre (2008) | Turkey | RC | 2001-2007 | 144 | - | - | - | - | 6 | - | - | - | - | - | - | No | 0 | - |
| Emre (2016) | Turkey | RC | - | 237 | 49.6 | (20-68) | 29 | 208 | - | - | - | - | - | - | - | Yes | - | - |
| Enomoto (2014) | Japan | RC | 2008-2010 | 844 | 45.4 | 17.2 | 153 | 691 | - | - | - | - | - | - | - | Yes | - | - |
| Erbil (2007) | Turkey | CC | 1990-2005 | 3250 | 47 | 14 | 378 | 2872 | 6 | - | - | - | - | - | - | Yes | - | - |
| Erçetin (2019) | Turkey | PC | 2008-2016 | 748 | 47.8 | 13 | 130 | 665 | 12 | 398/397 | - | Medtronic | - | - | - | No | - | - |
| Farizon (2017) | France | RC | 2012-2015 | 195 | 53.4 | (14-88) | 34 | 161 | 12 | 195/0 | - | Medtronic | - | - | - | Yes | - | - |
| Fassari (2024) | Italy | RC | - | 300 | 48.6 | 11.9 | 121 | 179 | - | 150/150 | - | NIM Medtronic | - | 100 | - | No | - | - |
| Fei (2022) | China | RC | 2013-2018 | 106 | 32.25 | 6.83 | - | - | - | 54/52 | - | NIM Medtronic | - | - | - | No | - | - |
| Fiorelli (2021) | Brazil | RC | 2006-2018 | 239 | 50.8 | 14.82 | 39 | 200 | - | - | - | - | - | - | - | No | - | - |
| Formanez (2016) | Philippines | RC | 2009-2014 | 237 | 41 | (20-65) | 74 | 163 | 6 | 109/128 | - | - | - | - | - | No | - | - |
| Frattini (2010) | Italy | RC | - | 152 | 40.6 | (19-77) | 67 | 85 | 12 | 76/76 | - | NIM Medtronic | - | - | - | No | - | 36 |
| Gao (2015) | China | RC | 2012-2013 | 137 | 32.02 | 8.32 | 2 | 135 | 6 | - | - | - | - | - | - | No | - | - |
| Giulea (2015) | Romania | PC | 2006-2013 | 100 | - | - | - | - | 6 | - | - | - | - | - | - | No | - | - |
| Giulea (2019) | Romania | RC | 2012-2015 | 90 | 54.5 | (25-83) | 17 | 73 | 12 | - | - | - | - | - | - | No | - | 21.1 |
| Godballe (2014) | Denmark | RC | 2001-2008 | 6859 | 50 | (6-100) | 1431 | 5428 | - | - | - | - | 8 | - | - | Yes | - | - |
| Grabovac (2013) | Croatia | RCT | - | 229 | 53 | (18-84) | 24 | 205 | - | - | - | - | - | - | - | No | - | - |
| Gremillion (2012) | USA | RC | 2007-2010 | 119 | - | - | - | - | - | 31/88 | - | - | - | - | - | No | - | - |
| Gunn (2020) | USA | RC | 2016-2017 | 11370 | 53 | (41-63) | 2476 | 8894 | 1 | 7031/4230 | - | - | 0.4 | - | - | No | - | - |
| Gür (2019) | Turkey | RC | 2014-2017 | 456 | 52.8 | (18-82) | 106 | 350 | - | 456/0 | - | - | - | - | - | No | - | - |
| Gurrado (2016) | Italy, France, UK | RC | 2009-2013 | 8908 | 51.1 | 13.6 | 2306 | 6602 | 6 | - | - | - | - | Selected cases | - | Yes | - | - |
| Gutierrez-Alvarez (2023) | UAS | RC | 2019-2022 | 218 | - | - | 39 | 179 | - | 150/68 | - | NIM Medtronic 3.0 | - | - | - | No | - | - |
| Haddadin (2023) | Jordan | RC | 2019-2022 | 255 | 39 | (21-59) | 74 | 181 | - | - | - | - | - | - | - | Yes | - | - |
| Hamilton (2019) | UK | RC | 2014-2016 | 256 | - | 27-86 | 27 | 169 | - | - | - | APS Medtronic | - | - | - | No | - | - |
| Hammad (2016) | USA | PC | 2013-2015 | 301 | 51.95 | 13.98 | 63 | 238 | - | - | - | - | - | - | - | Yes | 3.35 | - |
| Hardman (2015) | UK | RC | 1993-2013 | 1657 | 47.5 | 15.6 | 341 | 1316 | 6 | - | - | - | 11.39 | - | - | No | - | - |
| Hasin (2020) | Iraq | RC | 2017-2019 | 122 | 44.7 | 7 | - | - | - | - | - | - | - | 100 | 48 | No | - | - |
| Hei (2016a) | China | RCT | 2012-2014 | 70 | 47.5 | 9.9 | 16 | 54 | 6 | 41/43 | Intermittent | NIM Medtronic 2.0 | 100 | - | - | Yes | - | 68.57 |
| Hei (2016b) | China | RC | 2009-2011 | 97 | 45.35 | 11.23 | 19 | 78 | - | 46/51 | Intermittent | NIM Medtronic 2.0 | - | - | - | No | - | - |
| Hindosh (2011) | Iraq | PC | 2008-2010 | 200 | 33 | 2 | 25 | 175 | 6 | - | - | - | - | - | - | No | - | - |
| Hirsch (2014) | Israel | RC | 2001-2010 | 161 | 55.9 | 13.8 | 23 | 138 | 12 | - | - | - | 3.1 | - | - | No | - | - |
| Hirunwiwatkul (2013) | Thailand | RCT | - | 40 | 44.6 | 12 | 3 | 37 | 3 | - | - | - | - | 100 | - | No | - | - |
| Hoff (2024) | Norway | PC | - | 154 | 49.5 | 12.8 | 15 | 124 | 18 | - | - | - | - | 100 | - | No | - | - |
| Hu (2016) | China | RC | 2003-2014 | 5559 | 55 | (9-87) | 714 | 4845 | 6 | 0/5559 | - | - | 3.39 | - | - | No | - | 3.9 |
| Huang (2015) | Taiwan | RC | 2001-2003 | 3428 | - | 20-79 | 549 | 2879 | - | - | - | - | - | - | - | No | - | - |
| Idris (2013) | Sudan | PC | 2009-2012 | 82 | 42.8 | 8.4 | 13 | 69 | - | - | - | - | - | - | - | No | - | - |
| Iqbal (2016) | Pakistan | RCT | 2013-2014 | 150 | - | 13-60 | 53 | 97 | - | 75/75 | - | - | - | 100 | 24-72 | No | - | - |
| Jawad (2018) | Baghdad | PC | 2012-2016 | 132 | 37.35 | 8.37 | 47 | 85 | 6 | 64/54 | - | - | - | - | - | Yes | - | - |
| Joliat (2017) | Switzerland | RC | 2005-2013 | 451 | 50 | 43-63 | 12 | 51 | 12 | 8/55 | - | - | 12.69 | - | - | No | - | - |
| Jonas (2006) | France | PC | 1999-2004 | 937 | 50.8 | (24-83) | - | - | 12 | - | - | Neurosign 100 | 5.4 | - | - | No | - | - |
| Kai (2017) | China | RC | 2013-2016 | 522 | 65.66 | 0.3 | 122 | 430 | - | 340/212 | - | NIM Medtronic 3.0 | - | - | - | Yes | - | 10.17 |
| Karpathiotakis (2022) | Italy | PC | 2018-2020 | 100 | 55 | (43-65) | 17 | 83 | 6 | 50/50 | Intermittent | NIM Medtronic 3.0 | - | - | - | No | - | - |
| Khan (2022) | Pakistan | CS | 2020-2021 | 70 | 44.43 | - | 18 | 52 | 6 | 57/23 | - | - | - | - | - | Yes | - | - |
| Kim (2021) | USA | RC | 2016-2018 | 17610 | 52 | 15 | 3904 | 13706 | - | 11248/6362 | - | - | - | - | - | No | - | 24.3 |
| Kumar (2019) | Pakistan | RC | 2014-2018 | 120 | 45.89 | 4.17 | 12 | 108 | 6 | - | - | - | 3.33 | - | - | No | - | - |
| Kuryga (2021) | Poland | RC | 2005-2012 | 1235 | 49.5 | - | 169 | 1065 | 36 | 182/1052 | - | CLEO nerve monitor | - | - | - | Yes | - | - |
| Landerholm (2014) | Sweden | PC | 1984-2011 | 973 | 54.7 | 16.1 | 242 | 1080 | 12 | 0/973 | - | - | 4.9 | - | - | No | - | - |
| Lenay-Pinon (2021) | France | RC | 2013-2019 | 1026 | 53 | (18-81) | 266 | 760 | 12 | - | - | C2 NerveMonitor (Inomed) | - | - | - | Yes | - | - |
| Leow (2020) | Singapore | RC | 2014-2018 | 261 | 49.2 | 12.5 | 68 | 193 | - | 108/153 | - | NIM Medtronic 3.0 | 8 | - | - | Yes | - | 4.2 |
| Ling (2020) | China | RC | 2012-2017 | 1696 | 52 | (40-59) | 280 | 753 | 6 | 1104/592 | Intermittent | NIM Medtronic 3.0 | 5.13 | - | - | No | - | - |
| Liu (2020) | China | RC | 2017-2019 | 2350 | 51.9 | 13.3 | 371 | 1887 | 6 | 2350/0 | Intermittent | NIM Medtronic 3.0 | - | - | - | No | - | - |
| Liu (2021) | China | RC | 2012-2019 | 415 | 35.45 | (19-48) | 1 | 404 | 6 | 415/0 | - | NIM Medtronic 3.0 | - | - | - | Yes | - | - |
| Machens (2018) | Germany | RC | 1994-2017 | 167 | 6.9 | - | 78 | 89 | 6 | 167/0 | - | - | - | - | - | No | - | 34.73 |
| Maeda (2006) | Japan | RC | 2000-2004 | 63 | 34.1 | - | - | - | 26 | - | - | - | - | - | - | No | - | - |
| Mahoney (2021) | USA | RC | 2016-2017 | 11552 | >=65 | - | 2514 | 9038 | - | 7130/4422 | - | - | - | - | 28.21 | No | - | 28.28 |
| Maksimoski (2022) | USA | RC | 2012-2017 | 1025 | 13.9 | - | 228 | 797 | - | 795/230 | - | - | - | - | - | No | - | - |
| Marin Arteaga (2018) | Switzerland | RC | 2012-2016 | 1001 | 55 | 16.8 | 35 | 66 | 6 | 1001/0 | Continuous | NIM Medtronic 3.0 | - | - | - | No | - | 4 |
| Maurer (2019) | Germany | RCT | - | 205 | 43 | (18-84) | 40 | 165 | - | - | - | - | - | - | - | No | - | - |
| Maurer (2020) | Germany | RC | 2017-2019 | 1808 | 44 | (14-80) | 330 | 1478 | - | 3409/16 | Intermittent / Continuous | - | - | - | - | No | 1 | - |
| Messenbaeck (2018) | Austria | RC | - | 246 | 45.6 | (21-73) | 29 | 217 | - | 246/0 | - | Medtronic | - | - | - | No | - | - |
| Mirallié (2018) | France | PC | 2012-2014 | 1328 | 51.2 | (18-80) | 267 | 1061 | 6 | 807/521 | - | Medtronic | - | Surgeon's discretion | - | No | - | 2.19 |
| Mismar (2024) | Jordan | RC | 2010-2016 | 345 | - | - | - | - | 6 | - | - | - | 0.57 | - | - | No | - | - |
| Mizuno (2019) | Japan | RC | 2008-2017 | 5084 | 57.7 | 14.6 | 1528 | 4276 | 1 | 849/4955 | - | - | - | - | - | No | - | 36.5 |
| Mobayen (2015) | Iran | RC | 2009-2011 | 173 | 44.8 | 11.9 | 28 | 145 | 20 | - | - | - | - | - | - | No | - | - |
| Mohammad (2022) | Kuwait | RC | 2016-2019 | 197 | 49 | 23-85 | 71 | 126 | 6 | 171/26 | - | NIM Medtronic 3.0 | - | - | - | Yes | - | 66.49 |
| Molinari (2015) | Brazil | RC | 1997-2014 | 3411 | 48.7 | 15.5 | 404 | 3007 | - | - | - | - | - | 100 | - | No | - | 1.4 |
| Moreira (2020) | Australia | RC | 2010-2017 | 1003 | - | - | 220 | 783 | - | 1003/0 | - | NIM | - | - | - | Yes | - | - |
| Muhammad (2021) | Malaysia | RCT | 2016 | 25 | 54.2 | - | 7 | 33 | - | 20/20 | - | NIM Medtronic 3.0 | - | - | - | No | - | - |
| Mulita (2022) | Greece | RC | 2015-2020 | 306 | 54.25 | 10.9 | 68 | 238 | - | - | - | - | - | - | - | No | - | - |
| Nagaoka (2022) | Japan | RC | 2016-2020 | 100 | 36.2 | - | 1 | 99 | 6 | 25/75 | - | - | - | - | - | No | - | - |
| Nagaty (2023) | Egypt | RCT | 2018-2020 | 80 | 38.25 | (21-61) | 22 | 58 | - | - | - | - | - | 100 | - | Yes | - | - |
| Nayyar (2020) | India | RC | 2017-2019 | 228 | - | - | 150 | 250 | - | 150/250 | - | - | 33.8 | 100 | - | No | - | - |
| Ngo (2023) | Vietnam | RC | 2020-2022 | 27 | 16.3 | 2 | 3 | 24 | - | - | - | - | - | - | - | No | - | 7.4 |
| Nguyen (2021) | Vietnam | PC | 2018-2019 | 101 | 40 | 11.1 | 7 | 94 | - | - | - | - | - | - | - | No | - | - |
| Ozbas (2005) | Turkey | RC | 1994-2000 | 750 | 43.66 | - | - | - | 18 | - | - | - | - | - | - | No | - | - |
| Paek (2022) | Korea | RC | 2013-2014 | 315 | 42.45 | 9.9 | 70 | 245 | 6 | 315/0 | - | NIM Medtronic 3.0 | - | - | - | No | - | 17.36 |
| Palmer (2005) | USA | RC | 1987-1999 | 36 | - | - | 7 | 29 | - | - | - | - | - | - | - | No | - | - |
| Pantvaidya (2018) | India | RC | 2013-2014 | 152 | 37.5 | - | 63 | 89 | 12 | - | - | - | 28 | - | - | Yes | - | - |
| Papavramidis (2010) | Greece | RCT | 2008 | 90 | 48.99 | 12.59 | 13 | 77 | - | - | - | - | - | - | - | Yes | - | - |
| Park (2019) | Korea | RC | 2002-2016 | 396 | 49.5 | 18-81 | 72 | 324 | - | - | - | - | - | - | - | No | - | - |
| Pei (2021) | China | RC | 2010-2020 | 109 | 49.56 | 14.98 | 48 | 61 | - | 65/44 | - | NIM Medtronic 3.0 | 100 | - | - | Yes | - | - |
| Pelizzo (2014) | Italy | RC | 2006-2010 | 233 | - | - | - | - | - | - | - | - | 100 | - | - | No | - | - |
| Pergel (2014) | Turkey | RC | 2009-2011 | 456 | 49.25 | 11 | 127 | 329 | - | - | - | - | - | in some cases | - | No | - | - |
| Périé (2013) | France | PC | 2007-2011 | 100 | 47.1 | 16-81 | 19 | 81 | 6 | - | - | Neurosign 400 | 7 | 100 | 24 | Yes | - | - |
| Piccoli (2019) | Italy | RC | 2010-2018 | 449 | 44.1 | 12.1 | 62 | 387 | - | - | - | - | - | - | - | No | - | - |
| Pieracci (2007) | USA | RC | 1998-2004 | 33930 | 49.4 | 15.4 | 6373 | 27557 | - | - | - | - | - | - | - | No | - | - |
| Porseyedi (2012) | Iran | RC | 2005-2011 | 566 | 40.26 | - | 124 | 442 | - | 337/229 | - | - | - | - | - | No | - | - |
| Prokopakis (2013) | Greece | RC | 2004-2011 | 97 | 61 | 47-75 | 20 | 77 | - | - | - | NIM Medtronic | 100 | - | - | Yes | - | - |
| Qu (2021) | China | RC | - | 134 | 45.1 | 9.7 | 37 | 97 | - | - | - | - | - | - | - | No | - | - |
| Rafferty (2007) | Canada | RC | 1994-2004 | 350 | 46.55 | - | 62 | 288 | 12 | - | - | - | - | - | - | No | - | - |
| Rasool (2020) | Pakistan | RC | 2013-2018 | 409 | 41.5 | 12.7 | 85 | 324 | - | - | - | - | - | - | - | No | - | - |
| Raval (2009) | USA | RC | 2000-2007 | 31 | 12.2 | (5-17) | 6 | 25 | 6 | 23/8 | - | Medtronic | - | - | - | No | - | - |
| Razavi (2018) | USA | RC | 2016-2017 | 27 | 41.3 | 12.2 | 4 | 23 | 3 | - | - | NIM | - | - | - | No | - | - |
| Riju (2019) | India | PC | 2017 | 48 | 43.9 | 13.4 | 12 | 36 | 6 | - | - | - | - | - | - | No | - | - |
| Ríos-Zambudio (2004) | Spain | PC | 1996-2001 | 301 | 48 | 14 | 33 | 268 | 12 | - | - | - | - | - | - | No | - | - |
| Ritter (2021) | Israel | RC | 2001-2019 | 113 | 13.5 | 3.9 | 29 | 84 | 12 | - | - | NIM 2.0/3.0 | - | - | - | No | - | 19 |
| Robertson (2004) | USA | RC | 1999-2002 | 165 | 44.4 | - | 54 | 182 | - | 82/83 | - | NIM 2.0 | 3 | - | - | No | - | - |
| Rosato (2004) | Italy | RC | 1995-2001 | 14934 | 49 | - | 3131 | 11799 | - | - | - | - | 6 | - | - | No | - | - |
| Rossi (2022) | Italy | RC | 2012-2020 | 541 | 36.6 | 16-73 | 4 | 537 | 6 | - | - | - | - | - | - | No | - | - |
| Rudolph (2014) | France | RC | 1991-2006 | 494 | 39 | - | 41 | 453 | 6 | 494/0 | - | - | 100 | - | - | Yes | - | - |
| Russell (2021) | USA | RC | 2017-2020 | 533 | 44 | (10-84) | 90 | 443 | 6 | 533/0 | - | NIM 3.0 | - | - | - | Yes | - | - |
| Saint Marc (2007) | Italy | RCT | 2004 | 200 | 49.5 | 11.3 | 36 | 164 | - | - | - | - | - | - | - | No | - | - |
| Sajid (2016) | Pakistan | RC | 2013-2014 | 177 | 44.76 | 10.95 | - | - | - | - | - | - | - | - | - | Yes | - | - |
| Sanguinetti (2014) | Italy | RC | 2012 | 350 | - | - | - | - | - | 105/245 | - | - | - | - | - | No | - | - |
| Santosh (2014) | India | RC | 2009-2013 | 105 | - | - | - | - | 6 | - | - | - | - | - | - | Yes | - | - |
| Sarkis (2017) | Australia | RC | 1990-2014 | 7406 | - | - | - | - | 3 | 7406/0 | - | - | - | - | - | No | - | - |
| Sartori (2008) | Italy | RCT | 2007 | 150 | 56 | 14 | 27 | 123 | 6 | - | - | - | - | 100 | 24 | Yes | - | - |
| Schietroma (2017) | Italy | RCT | 2012-2016 | 215 | 47.88 | 8.7 | 84 | 131 | 6 | - | - | - | - | 108/107 | - | No | - | - |
| Schneider (2019) | Austria | PC | 2012-2016 | 4707 | - | - | 1212 | 3495 | 12 | 4707/0 | - | - | - | - | - | Yes | 10.25 | - |
| Sena (2019) | Italy | RC | 2009-2018 | 237 | 52.7 | - | 89 | 199 | - | - | - | NIM 2.4 and 3.0 | - | - | - | No | - | - |
| Shakir (2016) | Pakistan | RC | 2012-2015 | 144 | - | - | 21 | 123 | - | - | - | - | - | - | - | No | - | - |
| Shen (2013) | China | RC | 2005-2011 | 5344 | 48.5 | (3-86) | 1418 | 3926 | 6 | - | - | - | 25.7 | - | - | No | - | - |
| Shindo (2007) | USA | RC | 1998-2005 | 684 | - | - | - | - | - | 671/372 | - | Medtronic | - | - | - | No | - | 17.9 |
| Sleptsov (2023) | Germany | PC | 2021-2022 | 259 | 45 | 13 | 49 | 212 | - | - | - | - | - | - | - | No | - | 30.61 |
| Snyder (2010) | USA | RC | 2003-2009 | 1242 | 57.3 | - | - | - | - | 1242/0 | - | - | - | - | - | No | - | 13 |
| Snyder (2013) | USA | RC | 2004-2011 | 1936 | 52 | - | 685 | 2750 | - | 3354/81 | - | NIM Medtronic | - | - | - | No | - | - |
| Sopiński (2017) | China | RCT | 2014-2016 | 80 | 57.95 | 9.35 | 4 | 76 | - | 27/53 | Intermittent | C2 NerveMonitor | - | - | - | Yes | - | - |
| Sreejayan (2017) | India | PC | 2008-2015 | 823 | - | - | 289 | 534 | 6 | - | - | - | - | - | - | No | - | - |
| Stevens (2012) | USA | PC | 2004-2008 | 91 | 48.45 | 12.9 | 37 | 54 | 6 | 39/52 | - | NIM Medtronic | - | - | - | No | - | - |
| Tabriz (2024) | Germany | RC | 2016-2020 | 1147 | 52 | (13-90) | 293 | 854 | - | 1147/0 | Intermittent | - | - | - | - | Yes | - | - |
| Testini (2014) | Italy | RC | 1999-2008 | 14993 | 55.8 | (17-86) | 4858 | 10135 | 12 | - | - | - | - | - | - | No | - | - |
| Tsuzuki (2019) | Japan | RC | 2010-2015 | 146 | 52 | - | 27 | 119 | 6 | - | - | - | - | - | - | No | - | - |
| Vasileiadis (2016) | Greece | RC | 2002-2012 | 2566 | 51.35 | 14.18 | 528 | 2028 | 12 | 1481/1075 | - | NIM 2.0 | - | - | - | Yes | - | - |
| Velayutham (2022) | India | PC | 2017-2019 | 84 | - | - | - | - | - | 84/0 | - | NIM Medtronic 3.0 | - | - | - | Yes | - | - |
| Veyseller (2011) | Turkey | PC | 2006-2009 | 195 | 44.7 | (14-79) | 34 | 161 | 26 | - | - | - | 6.15 | - | - | No | - | - |
| Vural (2021) | Turkey | PC | 2005-2011 | 241 | 46.5 | (17-75) | 51 | 189 | 6 | - | - | - | - | - | - | No | - | - |
| Waheed (2017) | Pakistan | CS | 2008-2014 | 104 | 48 | 8 | 20 | 84 | - | - | - | - | - | - | - | No | - | - |
| Wojtczak (2017) | Poland | PC | 2011-2014 | 632 | 53.94 | 13.87 | 117 | 515 | 6 | 236/396 | - | NIM 3.0 | 10.87 | - | - | No | - | - |
| Wu (2017) | Taiwan | PC | 2012-2014 | 323 | 50 | (16-83) | 63 | 260 | 6 | - | - | NIM 3.0 | - | - | - | No | - | - |
| Wu (2018) | USA | RC | 2006-2015 | 380 | 38.5 | 14.04 | 71 | 309 | - | 288/92 | - | NIM Medtronic | 18.2 | - | - | No | - | 87.66 |
| Xu (2023) | China | RC | 2015-2021 | 416 | 37.8 | 7.87 | - | - | 6 | 416/0 | Intermittent | NIM | - | - | - | No | 59.1 | 100 |
| Yu (2020) | China | RC | 2016-2017 | 93 | 50 | 24-78 | 22 | 71 | - | 93/0 | - | Medtronic | - | - | - | No | - | - |
| Yu (2021) | China | PC | 2014-2017 | 321 | 33.1 | 6.9 | 17 | 304 | - | - | - | - | - | - | - | No | - | - |
| Yuksekdag (2019) | Turkey | RC | 2014-2018 | 260 | 51 | 32-67 | - | - | 6 | - | - | NIM Response 3.0 | - | - | - | No | - | - |

A total of 199 studies were deemed eligible for data synthesis and were included in this meta-analytic study (10-18, 25-27, 35-221).YOP: year of publication; RC: retrospective cohort; PC: prospective cohort; RCT: randomized controlled trial; CC: case-control; CS: cross-sectional; UK: United Kingdom; USA: United States of America; SD: standard deviation; M: male; F: female; LN: lymph node; LND: lateral neck dissection; CND: central neck dissection; YOI: year of investigation; FU: follow-up; mo: month.
