# Supplementary material for: Prevalence and determinants of recurrent laryngeal nerve injury after thyroidectomy: a Systematic Review and meta-analysis
Source: Front Endocrinol (Lausanne). 2026 Apr 29;17:1764332. doi: 10.3389/fendo.2026.1764332 (PMC13167572; doi:10.3389/fendo.2026.1764332)
Supplement: Supplementary file 5 [file Table4.docx]

**Supplemental Table 4:** The methodological quality of included observational studies using the Newcastle Ottawa Scale

| Author (YOP) | Selection | | | | Comparability | | Outcome | | | Overall Rating |
| --- | --- | --- | --- | --- | --- | --- | --- | --- | --- | --- |
|  | Representativeness of the exposed cohort | Selection of the non-exposed cohort | Ascertainment of exposure | Demonstration that outcome of interest was not present at start of study | Design | Analysis | Assessment of outcome | Was follow-up long enough for outcomes to occur | Adequacy of follow-up of cohorts |  |
| Abdelhamid (2021) | Yes | Yes | Yes | Yes | Yes | No | Yes | No | No | Fair |
| Acun (2005) | Yes | Yes | Yes | Yes | Yes | No | Yes | Yes | Yes | Good |
| Afolabi (2016) | Yes | Yes | Yes | Yes | Yes | No | Yes | Yes | Yes | Good |
| Akici (2020) | Yes | Yes | Yes | Yes | Yes | No | Yes | Yes | Yes | Good |
| Akkari (2014) | No | Yes | Yes | Yes | Yes | No | Yes | No | No | Poor |
| Alesina (2012) | Yes | Yes | Yes | Yes | Yes | No | Yes | No | No | Fair |
| AlHakami (2019) | Yes | Yes | Yes | Yes | Yes | No | Yes | No | No | Fair |
| Alhan (2015) | Yes | Yes | Yes | Yes | Yes | No | Yes | No | No | Fair |
| Alharbi (2018) | Yes | Yes | Yes | Yes | Yes | No | Yes | No | No | Fair |
| Ali (2019) | Yes | Yes | Yes | Yes | Yes | No | Yes | No | No | Fair |
| Ali (2022) | Yes | Yes | Yes | Yes | Yes | No | Yes | No | No | Fair |
| Ali (2012) | Yes | Yes | Yes | Yes | Yes | No | Yes | Yes | Yes | Good |
| Almosallam (2020) | Yes | Yes | Yes | Yes | Yes | No | Yes | No | No | Fair |
| Alqahtani (2023) | Yes | Yes | Yes | Yes | Yes | No | Yes | No | No | Fair |
| AlSaiegh (2020) | Yes | Yes | Yes | Yes | Yes | No | Yes | Yes | Yes | Good |
| Ambe (2014) | Yes | Yes | Yes | Yes | Yes | No | Yes | No | No | Fair |
| Amer (2022) | Yes | Yes | Yes | Yes | Yes | No | Yes | No | No | Fair |
| Anuwong (2018) | Yes | Yes | Yes | Yes | Yes | No | Yes | Yes | Yes | Good |
| Arikan (2023) | Yes | Yes | Yes | Yes | Yes | No | Yes | No | No | Fair |
| Ay (2013) | Yes | Yes | Yes | Yes | Yes | No | Yes | No | No | Fair |
| Aygun (2022) | Yes | Yes | Yes | Yes | Yes | No | Yes | No | No | Fair |
| Ban (2014) | Yes | Yes | Yes | Yes | Yes | No | Yes | No | No | Fair |
| Barczynski (2012b) | Yes | Yes | Yes | Yes | Yes | No | Yes | No | No | Fair |
| Barczyński (2014) | Yes | Yes | Yes | Yes | Yes | No | Yes | No | No | Fair |
| Baud (2022) | Yes | Yes | Yes | Yes | Yes | No | Yes | No | No | Fair |
| Bawa (2021) | Yes | Yes | Yes | Yes | Yes | No | Yes | No | No | Fair |
| Benkhadoura (2017) | No | Yes | Yes | Yes | Yes | No | Yes | Yes | Yes | Fair |
| Bergenfelz (2008) | Yes | Yes | Yes | Yes | Yes | No | Yes | No | No | Fair |
| Bergenfelz (2016) | Yes | Yes | Yes | Yes | Yes | No | Yes | No | No | Fair |
| Bertelli (2021) | No | Yes | Yes | Yes | Yes | No | Yes | Yes | Yes | Fair |
| Bihain (2021) | Yes | Yes | Yes | Yes | Yes | No | Yes | No | No | Fair |
| Bryk (2024) | Yes | Yes | Yes | Yes | Yes | No | Yes | No | No | Fair |
| Bukarica (2022) | No | Yes | Yes | Yes | Yes | No | Yes | No | No | Poor |
| Calò (2014a) | Yes | Yes | Yes | Yes | Yes | No | Yes | Yes | Yes | Good |
| Calò (2014b) | Yes | Yes | Yes | Yes | Yes | No | Yes | No | No | Fair |
| Calò (2014c) | Yes | Yes | Yes | Yes | Yes | No | Yes | Yes | Yes | Good |
| Čelakovský (2011) | Yes | Yes | Yes | Yes | Yes | No | Yes | Yes | Yes | Good |
| Celik (2011) | Yes | Yes | Yes | Yes | Yes | No | Yes | No | No | Fair |
| Chan (2006) | Yes | Yes | Yes | Yes | Yes | No | Yes | No | No | Fair |
| Chaudhary (2007) | Yes | Yes | Yes | Yes | Yes | No | Yes | No | No | Fair |
| Chen (2022a) | Yes | Yes | Yes | Yes | Yes | No | Yes | Yes | Yes | Good |
| Chen (2021) | Yes | Yes | Yes | Yes | Yes | No | Yes | Yes | Yes | Good |
| Chen (2022b) | No | Yes | Yes | Yes | Yes | No | Yes | Yes | Yes | Fair |
| Chereau (2024) | Yes | Yes | Yes | Yes | Yes | No | Yes | No | No | Fair |
| Chiang (2004) | Yes | Yes | Yes | Yes | Yes | No | Yes | No | No | Fair |
| Chiang (2011) | Yes | Yes | Yes | Yes | Yes | No | Yes | No | No | Fair |
| Chohan (2019) | Yes | Yes | Yes | Yes | Yes | No | Yes | No | No | Fair |
| Chuang (2013) | No | Yes | Yes | Yes | Yes | No | Yes | Yes | Yes | Fair |
| De Palma (2016) | Yes | Yes | Yes | Yes | Yes | No | Yes | Yes | Yes | Good |
| Dedhia (2020) | Yes | Yes | Yes | Yes | Yes | No | Yes | Yes | Yes | Good |
| D'Orazi (2019) | Yes | Yes | Yes | Yes | Yes | No | Yes | No | No | Fair |
| Dralle (2004) | Yes | Yes | Yes | Yes | Yes | No | Yes | Yes | Yes | Good |
| Efremidou (2009) | Yes | Yes | Yes | Yes | Yes | No | Yes | Yes | Yes | Good |
| ElLabban (2009) | No | Yes | Yes | Yes | Yes | No | Yes | No | No | Poor |
| Emre (2008) | Yes | Yes | Yes | Yes | Yes | No | Yes | No | No | Fair |
| Emre (2016) | Yes | Yes | Yes | Yes | Yes | No | Yes | No | No | Fair |
| Enomoto (2014) | Yes | Yes | Yes | Yes | Yes | No | Yes | Yes | Yes | Good |
| Erbil (2007) | Yes | Yes | Yes | Yes | Yes | No | Yes | No | No | Fair |
| Erçetin (2019) | Yes | Yes | Yes | Yes | Yes | No | Yes | Yes | Yes | Good |
| Farizon (2017) | Yes | Yes | Yes | Yes | Yes | No | Yes | No | No | Fair |
| Fassari (2024) | Yes | Yes | Yes | Yes | Yes | No | Yes | Yes | Yes | Good |
| Fei (2022) | Yes | Yes | Yes | Yes | Yes | No | Yes | Yes | Yes | Good |
| Fiorelli (2021) | Yes | Yes | Yes | Yes | Yes | No | Yes | No | No | Fair |
| Formanez (2016) | Yes | Yes | Yes | Yes | Yes | No | Yes | No | No | Fair |
| Frattini (2010) | Yes | Yes | Yes | Yes | Yes | No | Yes | No | No | Fair |
| Gao (2015) | Yes | Yes | Yes | Yes | Yes | No | Yes | No | No | Fair |
| Giulea (2019) | No | Yes | Yes | Yes | Yes | No | Yes | Yes | Yes | Fair |
| Giulea (2015) | Yes | Yes | Yes | Yes | Yes | No | Yes | Yes | Yes | Good |
| Godballe (2014) | Yes | Yes | Yes | Yes | Yes | No | Yes | No | No | Fair |
| Gremillion (2012) | Yes | Yes | Yes | Yes | Yes | No | Yes | Yes | Yes | Good |
| Gunn (2020) | Yes | Yes | Yes | Yes | Yes | No | Yes | No | No | Fair |
| Gür (2019) | Yes | Yes | Yes | Yes | Yes | No | Yes | Yes | Yes | Good |
| Gurrado (2016) | Yes | Yes | Yes | Yes | Yes | No | Yes | Yes | Yes | Good |
| GutierrezAlvarez (2023) | Yes | Yes | Yes | Yes | Yes | No | Yes | Yes | Yes | Good |
| Haddadin (2023) | Yes | Yes | Yes | Yes | Yes | No | Yes | Yes | Yes | Good |
| Hamilton (2019) | Yes | Yes | Yes | Yes | Yes | No | Yes | Yes | Yes | Good |
| Hammad (2016) | Yes | Yes | Yes | Yes | Yes | No | Yes | No | No | Fair |
| Hardman (2015) | Yes | Yes | Yes | Yes | Yes | No | Yes | No | No | Fair |
| Hasin (2020) | Yes | Yes | Yes | Yes | Yes | No | Yes | No | No | Fair |
| Hei (2016b) | No | Yes | Yes | Yes | Yes | No | Yes | No | No | Poor |
| Hindosh (2011) | Yes | Yes | Yes | Yes | Yes | No | Yes | No | No | Fair |
| Hirsch (2014) | Yes | Yes | Yes | Yes | Yes | No | Yes | Yes | Yes | Good |
| Hoff (2024) | Yes | Yes | Yes | Yes | Yes | No | Yes | Yes | Yes | Good |
| Hu (2016) | Yes | Yes | Yes | Yes | Yes | No | Yes | Yes | Yes | Good |
| Huang (2015) | Yes | Yes | Yes | Yes | Yes | No | Yes | Yes | Yes | Good |
| Idris (2013) | Yes | Yes | Yes | Yes | Yes | No | Yes | No | No | Fair |
| Jawad (2018) | Yes | Yes | Yes | Yes | Yes | No | Yes | No | No | Fair |
| Joliat (2017) | Yes | Yes | Yes | Yes | Yes | No | Yes | No | No | Fair |
| Jonas (2006) | Yes | Yes | Yes | Yes | Yes | No | Yes | No | No | Fair |
| Kai (2017) | Yes | Yes | Yes | Yes | Yes | No | Yes | Yes | Yes | Good |
| Karpathiotakis (2022) | Yes | Yes | Yes | Yes | Yes | No | Yes | Yes | Yes | Good |
| Khan (2022) | Yes | Yes | Yes | Yes | Yes | No | Yes | Yes | Yes | Good |
| Kim (2021) | Yes | Yes | Yes | Yes | Yes | No | Yes | Yes | Yes | Good |
| Kumar (2019) | Yes | Yes | Yes | Yes | Yes | No | Yes | No | No | Fair |
| Kuryga (2021) | Yes | Yes | Yes | Yes | Yes | No | Yes | Yes | Yes | Good |
| Landerholm (2014) | Yes | Yes | Yes | Yes | Yes | No | Yes | No | No | Fair |
| LenayPinon (2021) | Yes | Yes | Yes | Yes | Yes | No | Yes | Yes | Yes | Good |
| Leow (2020) | Yes | Yes | Yes | Yes | Yes | No | Yes | Yes | Yes | Good |
| Ling (2020) | Yes | Yes | Yes | Yes | Yes | No | Yes | Yes | Yes | Good |
| Liu (2020) | Yes | Yes | Yes | Yes | Yes | No | Yes | No | No | Fair |
| Liu (2021) | Yes | Yes | Yes | Yes | Yes | No | Yes | Yes | Yes | Good |
| Machens (2018) | Yes | Yes | Yes | Yes | Yes | No | Yes | Yes | Yes | Good |
| Maeda (2006) | No | Yes | Yes | Yes | Yes | No | Yes | Yes | Yes | Fair |
| Mahoney (2021) | Yes | Yes | Yes | Yes | Yes | No | Yes | Yes | Yes | Good |
| Maksimoski (2022) | Yes | Yes | Yes | Yes | Yes | No | Yes | Yes | Yes | Good |
| Marin Arteaga (2018) | Yes | Yes | Yes | Yes | Yes | No | Yes | No | No | Fair |
| Maurer (2020) | Yes | Yes | Yes | Yes | Yes | No | Yes | No | No | Fair |
| Messenbaeck (2018) | Yes | Yes | Yes | Yes | Yes | No | Yes | Yes | Yes | Good |
| Mirallié (2018) | Yes | Yes | Yes | Yes | Yes | No | Yes | No | No | Fair |
| Mismar (2024) | Yes | Yes | Yes | Yes | Yes | No | Yes | No | No | Fair |
| Mizuno (2019) | Yes | Yes | Yes | Yes | Yes | No | Yes | No | No | Fair |
| Mobayen (2015) | Yes | Yes | Yes | Yes | Yes | No | Yes | No | No | Fair |
| Mohammad (2022) | Yes | Yes | Yes | Yes | Yes | No | Yes | Yes | Yes | Good |
| Molinari (2015) | Yes | Yes | Yes | Yes | Yes | No | Yes | Yes | Yes | Good |
| Moreira (2020) | Yes | Yes | Yes | Yes | Yes | No | Yes | Yes | Yes | Good |
| Mulita (2022) | Yes | Yes | Yes | Yes | Yes | No | Yes | Yes | Yes | Good |
| Nagaoka (2022) | Yes | Yes | Yes | Yes | Yes | No | Yes | Yes | Yes | Good |
| Nayyar (2020) | Yes | Yes | Yes | Yes | Yes | No | Yes | No | No | Fair |
| Ngo (2023) | No | Yes | Yes | Yes | Yes | No | Yes | No | No | Poor |
| Nguyen (2021) | Yes | Yes | Yes | Yes | Yes | No | Yes | No | No | Fair |
| Ozbas (2005) | Yes | Yes | Yes | Yes | Yes | No | Yes | No | No | Fair |
| Paek (2022) | Yes | Yes | Yes | Yes | Yes | No | Yes | No | No | Fair |
| Palmer (2005) | No | Yes | Yes | Yes | Yes | No | Yes | Yes | Yes | Fair |
| Pantvaidya (2018) | Yes | Yes | Yes | Yes | Yes | No | Yes | No | No | Fair |
| Park (2019) | Yes | Yes | Yes | Yes | Yes | No | Yes | No | No | Fair |
| Pei (2021) | Yes | Yes | Yes | Yes | Yes | No | Yes | No | No | Fair |
| Pelizzo (2014) | Yes | Yes | Yes | Yes | Yes | No | Yes | No | No | Fair |
| Pergel (2014) | Yes | Yes | Yes | Yes | Yes | No | Yes | Yes | Yes | Good |
| Périé (2013) | Yes | Yes | Yes | Yes | Yes | No | Yes | Yes | Yes | Good |
| Piccoli (2019) | Yes | Yes | Yes | Yes | Yes | No | Yes | Yes | Yes | Good |
| Pieracci (2007) | Yes | Yes | Yes | Yes | Yes | No | Yes | No | No | Fair |
| Porseyedi (2012) | Yes | Yes | Yes | Yes | Yes | No | Yes | Yes | Yes | Good |
| Prokopakis (2013) | No | Yes | Yes | Yes | Yes | No | Yes | No | No | Poor |
| Qu (2021) | Yes | Yes | Yes | Yes | Yes | No | Yes | No | No | Fair |
| Rafferty (2007) | Yes | Yes | Yes | Yes | Yes | No | Yes | No | No | Fair |
| Rasool (2020) | Yes | Yes | Yes | Yes | Yes | No | Yes | No | No | Fair |
| Raval (2009) | No | Yes | Yes | Yes | Yes | No | Yes | No | No | Poor |
| Razavi (2018) | No | Yes | Yes | Yes | Yes | No | Yes | Yes | Yes | Fair |
| Riju (2019) | No | Yes | Yes | Yes | Yes | No | Yes | No | No | Poor |
| RíosZambudio (2004) | Yes | Yes | Yes | Yes | Yes | No | Yes | Yes | Yes | Good |
| Ritter (2021) | Yes | Yes | Yes | Yes | Yes | No | Yes | No | No | Fair |
| Robertson (2004) | Yes | Yes | Yes | Yes | Yes | No | Yes | No | No | Fair |
| Rosato (2004) | Yes | Yes | Yes | Yes | Yes | No | Yes | No | No | Fair |
| Rossi (2022) | Yes | Yes | Yes | Yes | Yes | No | Yes | No | No | Fair |
| Rudolph (2014) | Yes | Yes | Yes | Yes | Yes | No | Yes | No | No | Fair |
| Russell (2021) | Yes | Yes | Yes | Yes | Yes | No | Yes | Yes | Yes | Good |
| Sajid (2016) | Yes | Yes | Yes | Yes | Yes | No | Yes | No | No | Fair |
| Sanguinetti (2014) | Yes | Yes | Yes | Yes | Yes | No | Yes | Yes | Yes | Good |
| Santosh (2014) | Yes | Yes | Yes | Yes | Yes | No | Yes | Yes | Yes | Good |
| Sarkis (2017) | Yes | Yes | Yes | Yes | Yes | No | Yes | Yes | Yes | Good |
| Schneider (2019) | Yes | Yes | Yes | Yes | Yes | No | Yes | Yes | Yes | Good |
| Sena (2019) | Yes | Yes | Yes | Yes | Yes | No | Yes | Yes | Yes | Good |
| Shakir (2016) | Yes | Yes | Yes | Yes | Yes | No | Yes | Yes | Yes | Good |
| Shen (2013) | Yes | Yes | Yes | Yes | Yes | No | Yes | No | No | Fair |
| Shindo (2007) | Yes | Yes | Yes | Yes | Yes | No | Yes | No | No | Fair |
| Sleptsov (2023) | Yes | Yes | Yes | Yes | Yes | No | Yes | No | No | Fair |
| Snyder (2010) | Yes | Yes | Yes | Yes | Yes | No | Yes | Yes | Yes | Good |
| Snyder (2013) | Yes | Yes | Yes | Yes | Yes | No | Yes | Yes | Yes | Good |
| Sreejayan (2017) | Yes | Yes | Yes | Yes | Yes | No | Yes | Yes | Yes | Good |
| Stevens (2012) | No | Yes | Yes | Yes | Yes | No | Yes | Yes | Yes | Fair |
| Tabriz (2024) | Yes | Yes | Yes | Yes | Yes | No | Yes | Yes | Yes | Good |
| Testini (2014) | Yes | Yes | Yes | Yes | Yes | No | Yes | No | No | Fair |
| Tsuzuki (2019) | Yes | Yes | Yes | Yes | Yes | No | Yes | No | No | Fair |
| Vasileiadis (2016) | Yes | Yes | Yes | Yes | Yes | No | Yes | No | No | Fair |
| Velayutham (2022) | No | Yes | Yes | Yes | Yes | No | Yes | No | No | Poor |
| Veyseller (2011) | Yes | Yes | Yes | Yes | Yes | No | Yes | Yes | Yes | Good |
| Vural (2021) | Yes | Yes | Yes | Yes | Yes | No | Yes | Yes | Yes | Good |
| Waheed (2017) | Yes | Yes | Yes | Yes | Yes | No | Yes | No | No | Fair |
| Wojtczak (2017) | Yes | Yes | Yes | Yes | Yes | No | Yes | Yes | Yes | Good |
| Wu (2017) | Yes | Yes | Yes | Yes | Yes | No | Yes | No | No | Fair |
| Wu (2018) | Yes | Yes | Yes | Yes | Yes | No | Yes | Yes | Yes | Good |
| Xu (2023) | Yes | Yes | Yes | Yes | Yes | No | Yes | Yes | Yes | Good |
| Yu (2020) | No | Yes | Yes | Yes | Yes | No | Yes | Yes | Yes | Fair |
| Yu (2021) | Yes | Yes | Yes | Yes | Yes | No | Yes | Yes | Yes | Good |
| Yuksekdag (2019) | Yes | Yes | Yes | Yes | Yes | No | Yes | Yes | Yes | Good |

YOP: year of publication.
